# Thoracic and cardiovascular surgery in Japan during 2013: Annual report by The Japanese Association for Thoracic Surgery

**DOI:** 10.1007/s11748-015-0590-3

**Published:** 2015-11-12

**Authors:** Committee for Scientific Affairs, The Japanese Association for Thoracic Surgery, Munetaka Masuda, Hiroyuki Kuwano, Meinoshin Okumura, Hirokuni Arai, Shunsuke Endo, Yuichiro Doki, Junjiro Kobayashi, Noboru Motomura, Hiroshi Nishida, Yoshikatsu Saiki, Fumihiro Tanaka, Kazuo Tanemoto, Yasushi Toh, Hiroyasu Yokomise

**Affiliations:** 1 Tokyo, Japan; 2 Department of Surgery, Yokohama City University, Yokohama, Japan; 3 Department of General Surgical Science, Division of Biosystem Medicine, Subdivision of Oncology, Course of Medical Sciences, Gunma University Graduate School of Medicine, Gunma, Japan; 4 Department of General Thoracic Surgery, Osaka University Graduate School of Medicine, Osaka, Japan; 5 Department of Cardiovascular Surgery, Tokyo Medical and Dental University Graduate School of Medical and Dental Sciences, Tokyo, Japan; 6 Department of Thoracic Surgery, Jichi Medical University, Tochigi, Japan; 7 Department of Gastroenterological Surgery, Osaka University Graduate School of Medicine, Osaka, Japan; 8 Department of Cardiovascular Surgery, National Cerebral and Cardiovascular Center, Osaka, Japan; 9 Department of Cardiovascular Surgery, Toho University, Sakura Medical Center, Chiba, Japan; 10 Department of Cardiovascular Surgery, The Heart Institute of Japan, Tokyo Women’s Medical University, Tokyo, Japan; 11 Division of Cardiovascular Surgery, Tohoku University Graduate School of Medicine, Miyagi, Japan; 12 Second Department of Surgery, University of Occupational and Environmental Health, Fukuoka, Japan; 13 Department of Cardiovascular Surgery, Kawasaki Medical School, Okayama, Japan; 14 Department of Gastroenterological Surgery, National Kyushu Cancer Center, Fukuoka, Japan; 15 Department of General Thoracic Surgery, Faculty of Medicine, Kagawa University, Kagawa, Japan

The Japanese Association for Thoracic Surgery has conducted annual surveys of thoracic surgery throughout Japan since 1986 to determine the statistics regarding the number of procedures according to operative category. Here, we have summarized the results from our annual survey of thoracic surgery performed during 2013.

The incidence of hospital mortality was added to the survey to determine the nationwide status, which has contributed to the Japanese surgeons to understand the present status of thoracic surgery in Japan and to make progress to improve operative results by comparing their work with those of others. The Association was able to gain a better understanding of present problems as well as future prospects, which has been reflected to its activity including education of its members. Thirty-day mortality (so called “operative mortality”) is defined as death within 30 days of operation regardless of the patient’s geographic location and even though the patient had been discharged from the hospital.

Hospital mortality is defined as death within any time interval after an operation if the patient had not been discharged from the hospital. Hospital-to-hospital transfer is not considered discharge: transfer to a nursing home or a rehabilitation unit is considered hospital discharge unless the patient subsequently dies of complications of the operation. (The definitions of the Ad Hoc Liaison Committee for Standardizing Definitions of Prosthetic Heart Valve Morbidity of the Society of Thoracic Surgeons and the American Association for Thoracic Surgery (Edmunds et al. Ann Thorac Surg 1996;62:932–5; J Thorac Cardiovasc Surg 1996;112:708–11).

Thoracic surgery was classified into three categories—cardiovascular, general thoracic, and esophageal surgery—and the patient data were examined and analyzed for each group. Access to the computerized data is offered to all members of this Association. We honor and value all member’s continued kind support and contributions (Tables 1, 2).

**Table 1:** **Table 1** Questionnaires sent out and received back by the end of December 2014

	Sent out	Returned	Response rate (%)
(A) Cardiovascular surgery	602	589	97.8
(B) General thoracic surgery	793	761	96.0
(C) Esophageal surgery	577	559	96.9

**Table 2:** **Table 2** Categories subclassified according to the number of operations performed

Number of operations performed	Category	
	Cardiovascular surgery	General thoracic surgery
0	44	34
1–24	44	101
25–49	92	125
50–99	172	210
100–149	86	124
150–199	56	83
≧200	95	85
Total	589	762

Number of operations performed	Esophageal surgery	
0	84	
1–4	96	
5–9	84	
10–19	109	
20–29	56	
30–39	35	
40–49	26	
≧50	69	
Total	559	

## Abstract of the survey

We sent out survey questionnaire forms to the departments of each category in all 1535 institutions (602 cardiovascular, 793 general thoracic and 577 esophageal) nationwide in early April 2014. The response rates in each category by the end of December 2014 were 97.8, 96.0, and 96.9 %, respectively. This high response rate has been keep throughout recent survey, and more than 96 % response rate in all fields in 2013 survey has to be congratulated.

## 2013 Final report

### (A) Cardiovascular surgery

First, we are very pleased with the high response rate to our survey of cardiovascular surgery (97.8 %), which definitely enhances the quality of this annual report. We very much appreciate the enormous effort put into completing the survey at each participating institution.

**Fig. 1:**
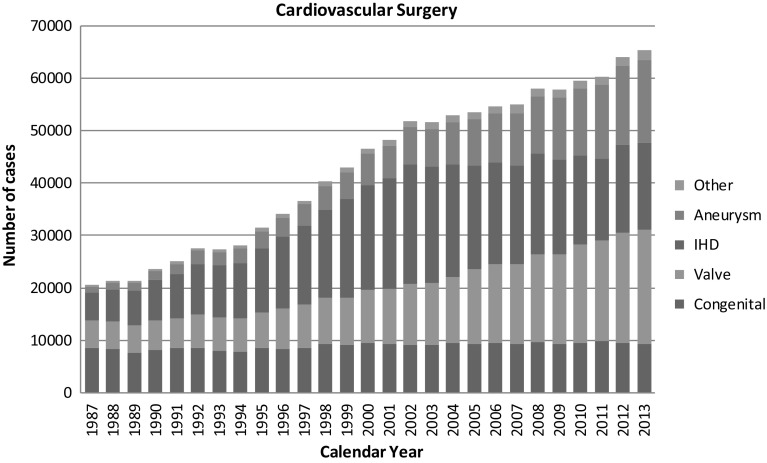
Cardiovascular surgery. *IHD* ischemic heart disease

Figure 1 shows the development of cardiovascular surgery in Japan over the last 27 years. Aneurysm surgery includes only operations for thoracic and thoracoabdominal aortic aneurysm. Pacemaker implantation includes only trans-thoracic implantation and trans-venous implantation is excluded. The number of pacemaker and assist device implantation operations is not included in the total number of surgical operations. A total of 67,325 cardiovascular operations were performed at 589 institutions during 2013 alone and included 36 heart transplantations, which were re-started in 1999, and 1 heart and lung transplantation.

The number of operations for congenital heart disease (9366 cases) decreased slightly (2.0 %) compared with that of 2012 (9558 cases), while there was 2.1 % increase when compared with the data of 10 years ago (9168 cases in 2003). The number of operations for adult cardiac disease (21,758 cases in valvular heart disease, 15,757 cases in thoracic aortic aneurysm and 1871 cases for other procedures) increased compared with those of 2012 (4.0, 4.6 and 14.6 %, respectively) except for ischemic heart disease (16,752 cases,) which decreased 1.9 % of that in 2012. During the last 10 years, the numbers of operations for adult heart disease increased constantly except for that for ischemic heart disease (83.4 % increase in valvular heart disease, 25.4 % decrease in ischemic heart disease, 120.9 % increase in thoracic aortic aneurysm, and 45.7 % increase in other procedures compared those of 2003). The concomitant coronary artery bypass grafting procedure (CABG) is not included in ischemic heart disease but included in other categories such as valvular heart disease and thoracic aneurysm in our study; then, the number of CABG still remained over 20,000 cases per year (21,242 cases) in 2013, which is 87.8 % of that in 2003 (24,204 cases).

Data for individual categories are summarized in tables through 3 to 9.

**Table 3:** **Table 3** Congenital (total; 9366) (1) CPB (+) (total; 7150)

		Neonate				Infant				1–17 years				≧18 years				Total			
		Cases	30-day mortality		Hospital mortality	Cases	30-day mortality		Hospital mortality	Cases	30-day mortality		Hospital mortality	Cases	30-day mortality		Hospital mortality	Cases	30-day mortality		Hospital mortality
			Hospital	After discharge			Hospital	After discharge			Hospital	After discharge			Hospital	After discharge			Hospital	After discharge	
1	PDA	3	0	0	0	1	0	0	0	2	0	0	0	14	1 (7.1)	0	1 (7.1)	20	1 (5.0)	0	1 (5.0)
2	Coarctation (simple)	6	0	0	0	10	0	0	0	11	0	0	0	8	0	0	0	35	0	0	0
3	+VSD	59	1 (12.5)	0	0	50	1 (2.0)	0	2 (4.0)	9	0	0	0	2	0	0	0	120	1 (0.8)	0	2 (1.7)
4	+DORV	8	0	0	1 (12.5)	3	0	0	0	5	0	0	0	0	0	0	0	16	1 (6.3)	0	1 (6.3)
5	+AVSD	2	2 (100.0)	0	2 (100.0)	2	1 (50.0)	0	1 (50.0)	0	0	0	0	1	0	0	0	5	3 (60.0)	0	3 (60.0)
6	+TGA	6	0	0	0	2	0	0	0	0	0	0	0	0	0	0	0	8	0	0	0
7	+SV	8	0	0	0	6	0	0	0	2	0	0	0	0	0	0	0	16	0	0	0
8	+Others	9	0	0	0	2	0	0	0	5	0	0	0	3	0	0	0	19	0	0	0
9	Interrupt. of Ao (simple)	1	0	0	0	1	0	0	0	0	0	0	0	0	0	0	0	2	0	0	0
10	+VSD	30	2 (6.7)	0	2 (6.7)	24	1 (4.2)	0	1 (4.2)	5	0	0	0	2	0	0	0	61	3 (4.9)	0	3 (4.9)
11	+DORV	4	0	0	0	1	0	0	0	0	0	0	0	0	0	0	0	5	0	0	0
12	+Truncus	0	0	0	0	1	0	0	0	0	0	0	0	0	0	0	0	1	0	0	0
13	+TGA	0	0	0	0	0	0	0	0	1	0	0	0	0	0	0	0	1	0	0	0
14	+Others	2	0	0	0	9	1 (11.1)	0	1 (11.1)	4	0	0	0	0	0	0	0	15	1 (6.7)	0	1 (6.7)
15	Vascular ring	0	0	0	0	3	0	0	0	1	0	0	0	0	0	0	0	4	0	0	0
16	PS	0	0	0	0	10	0	0	0	22	1 (4.5)	0	0	3	0	0	0	35	1 (2.9)	0	0
17	PAIVS or critical PS	20	0	0	2 (10.0)	57	0	0	0	55	0	0	1 (1.8)	5	1 (20.0)	0	1 (20.0)	137	1 (0.7)	0	4 (2.9)
18	TAPVR	108	12 (11.1)	0	15 (13.9)	55	2 (4)	0	2 (3.6)	12	0	0	0	0	0	0	0	175	14 (8)	0	17 (10)
19	PAPVR ± ASD	8	0	0	0	13	0	0	0	46	0	0	0	28	0	0	0	95	0	0	0
20	ASD	35	0	2 (5.7)	0	65	0	0	0	676	0	0	0	545	0	0	2 (0.4)	1321	0	2 (0.15)	2 (0.2)
21	Cor triatriatum	0	0	0	0	12	0	0	0	5	0	0	0	2	0	0	0	19	0	0	0
22	AVSD (partial)	0	0	0	0	18	0	0	0	57	0	0	0	16	1 (6.3)	0	1 (6.3)	91	1 (1.1)	0	1 (1.1)
23	AVSD (complete)	1	0	0	0	90	0	0	0	75	1 (1.3)	0	1 (1.3)	2	0	0	0	168	1 (0.6)	0	1 (0.6)
24	+TOF or DORV	2	0	0	0	13	0	0	0	23	1 (4.3)	0	1 (4.3)	0	0	0	0	38	1 (2.6)	0	1 (2.6)
25	+Others	1	0	0	1 (100.0)	4	0	0	1 (25.0)	9	0	0	1 (11.1)	0	0	0	0	14	0	0	3 (21.4)
26	VSD (subarterial)	3	0	0	0	122	0	0	0	179	0	0	0	33	0	0	0	337	0	0	0
27	VSD (perimemb/muscular)	12	0	0	0	809	4 (0.5)	0	5 (0.6)	384	0	0	1 (0.3)	81	1 (1.2)	0	1 (1.2)	1286	5 (0.4)	0	7 (0.5)
28	VSD + PS	0	0	0	0	15	0	0	0	15	0	0	0	3	0	0	0	33	0	0	0
29	DCRV ± VSD	0	0	0	0	15	0	0	0	37	0	0	0	14	0	0	0	66	0	0	0
30	Aneurysm of sinus valsalva	0	0	0	0	0	0	0	0	2	0	0	0	33	1 (3.0)	0	1 (3.0)	35	1 (2.9)	0	1 (2.9)
31	TOF	8	0	0	0	206	1 (0.5)	0	2 (1.0)	235	2 (0.9)	0	4 (1.7)	40	0	0	1 (2.5)	489	3 (0.6)	0	7 (1.4)
32	PA + VSD	8	1 (12.5)	0	2 (25.0)	58	3 (5.2)	0	3 (5.2)	100	2 (2.0)	0	2 (2.0)	9	0	0	0	175	6 (3.4)	0	7 (4.0)
33	DORV	12	1 (8.3)	0	1 (8.3)	98	3 (3.1)	0	3 (3.1)	111	0	0	0	8	0	0	0	229	4 (1.7)	0	4 (1.7)
34	TGA (simple)	98	3 (3.1)	0	4 (4.1)	6	0	0	0	4	0	0	0	2	0	0	0	110	3 (2.7)	0	4 (3.6)
35	+VSD	31	1 (3.2)	0	2 (6.5)	18	1 (5.6)	0	1 (5.6)	7	0	0	0	2	0	0	0	58	2 (3.4)	0	3 (5.2)
36	VSD + PS	1	0	0	0	11	0	0	0	13	0	0	1 (7.7)	3	0	0	0	28	0	0	1 (3.6)
37	Corrected TGA	1	0	1 (100.0)	0	11	0	0	0	37	0	0	0	11	0	0	1 (9.1)	60	0	1 (1.7)	1 (1.7)
38	Truncus arteriosus	6	0	0	0	21	2 (9.5)	0	2 (9.5)	14	0	0	0	1	0	0	0	42	2 (4.8)	0	2 (4.8)
39	SV	31	5 (16.1)	0	9 (29.0)	193	10 (5.2)	0	15 (7.8)	287	4 (1.4)	0	6 (2.1)	16	0	0	0	527	19 (3.6)	0	30 (5.7)
40	TA	2	1 (50.0)	0	2 (100.0)	38	1 (2.6)	1 (2.6)	1 (2.6)	45	0	0	0	4	0	0	0	89	2 (2.2)	1 (1.1)	3 (3.4)
41	HLHS	38	7 (18.4)	0	11 (28.9)	109	3 (2.8)	0	9 (8.3)	72	0	0	0	0	0	0	0	219	10 (4.6)	0	20 (9.1)
42	Aortic valve lesion	4	0	0	0	21	0	0	2 (9.5)	89	0	0	0	27	0	0	0	141	0	0	2 (1.4)
43	Mitral valve lesion	5	0	0	1 (20.0)	38	1 (2.6)	0	1 (2.6)	62	1 (1.6)	0	1 (1.6)	11	0	0	0	116	2 (1.7)	0	3 (2.6)
44	Ebstein	16	1 (6.3)	0	3 (18.8)	16	1 (6.3)	0	1 (6.3)	25	0	0	0	21	0	0	0	78	2 (2.6)	0	4 (5.1)
45	Coronary disease	2	0	0	0	21	0	0	0	9	0	0	0	17	1 (5.9)	0	1 (5.9)	49	1 (2.0)	0	1 (2.0)
46	Others	11	0	0	2 (18.2)	29	3 (10.3)	0	4 (13.8)	47	1 (2.1)	0	2 (4.3)	11	0	0	0	98	4 (4.1)	0	8 (8.2)
47	Redo VSD	1	0	0	0	7	0	0	1 (14.3)	9	0	0	0	5	0	0	0	22	0	0	1 (4.5)
48	PS release	0	0	0	0	12	0	0	0	57	2 (3.5)	0	2 (3.5)	20	0	0	0	89	2 (2.2)	0	2 (2.2)
49	RV-PA conduit replace	2	0	0	0	5	0	0	0	60	0	0	0	35	0	0	0	102	0	0	0
50	Others	8	2 (25.0)	0	2 (25.0)	57	1 (1.8)	0	2 (3.5)	117	2 (1.7)	0	3 (2.6)	69	1 (1.4)	0	1 (1.4)	251	6 (2.4)	0	8 (3.2)
Total		613	39 (6.4)	3 (0.5)	62 (10.1)	2388	40 (1.7)	1 (0.04)	60 (2.5)	3042	17 (0.6)	0	26 (0.9)	1107	7 (0.6)	0	11 (1.0)	7150	103 (1.4)	4 (0.1)	159 (2.2)

Values in parenthesis represent mortality %

*CPB* cardiopulmonary bypass, *PDA* patient ductus arteriosus, *VSD* ventircular septal defect, *DORV* double outlet right ventricle, *AVSD* atrioventricular septal defect, *TGA* transposition of great arteries, *SV* single ventricle, *Interupt. of Ao.* interruption of aorta, *PS* pulmonary stenosis, *PA-IVS* pulmonary atresia with intact ventricular septum, *TAPVR* total anomalous pulmonary venous return, *PAPVR* partial anomalous pulmonary venous return, *ASD* atrial septal defect, *TOF* tetralogy of Fallot, *DCRV* double-chambered right ventricle, *TA* tricuspid atresia, *HLHS* hypoplastic left heart syndrome, *RV-PA* right ventricle-pulmonary artery

**Table 4:** (2) CPB (−) (total; 2216)

		Neonate				Infant				1–17 years				≧18 years				Total			
		Cases	30-day mortality		Hospital mortality	Cases	30-day mortality		Hospital mortality	Cases	30-day mortality		Hospital mortality	Cases	30-day mortality		Hospital mortality	Cases	30-day mortality		Hospital mortality
			Hospital	After discharge			Hospital	After discharge			Hospital	After discharge			Hospital	After discharge			Hospital	After discharge	
1	PDA	422	4 (0.9)	0	8 (1.9)	211	2 (0.9)	0	5 (2.4)	33	0	0	0	2	0	0	0	668	6 (0.9)	0	13 (1.9)
2	Coarctation (simple)	13	0	0	0	15	0	0	0	5	0	0	0	2	0	0	0	35	0	0	0
3	+VSD	47	1 (2.1)	0	2 (4.3)	23	0	0	0	1	0	0	0	0	0	0	0	71	1 (1.4)	0 (0.0)	2 (2.8)
4	+DORV	14	0	0	0	0	0	0	0	0	0	0	0	0	0	0	0	14	0	0	0
5	+AVSD	4	1 (25.0)	0	1 (25.0)	4	0	0	0	1	0	0	0	0	0	0	0	9	1 (11.1)	0	1 (11.1)
6	+TGA	6	0	0	0	1	0	0	0	0	0	0	0	0	0	0	0	7	0	0	0
7	+SV	12	0	0	0	3	0	0	0	0	0	0	0	0	0	0	0	15	0	0	0
8	+Others	3	0	0	0	4	1 (25.0)	0	1 (25.0)	0	0	0	0	0	0	0	0	7	1 (14.3)	0	1 (14.3)
9	Interrupt. of Ao (simple)	0	0	0	0	0	0	0	0	0	0	0	0	0	0	0	0	0	0	0	0
10	+VSD	27	1 (3.7)	0	1 (3.7)	0	0	0	0	0	0	0	0	0	0	0	0	27	1 (3.7)	0	1 (3.7)
11	+DORV	7	0	0	1 (14)	2	0	0	0	0	0	0	0	0	0	0	0	9	0	0	1 (11.1)
12	+Truncus	1	0	0	0	0	0	0	0	0	0	0	0	0	0	0	0	1	0	0	0
13	+TGA	2	0	0	0	0	0	0	0	0	0	0	0	0	0	0	0	2	0	0	0
14	+Others	8	1 (12.5)	0	2 (25.0)	1	0	0	0	0	0	0	0	0	0	0	0	9	1 (11.1)	0	2 (22.2)
15	Vascular ring	8	0	0	0	12	0	0	0	2	0	0	0	1	0	0	0	23	0	0	0
16	PS	0	0	0	0	2	0	0	0	2	0	0	0	0	0	0	0	4	0	0	0
17	PAIVS or critical PS	35	2 (5.7)	0	4 (11.4)	24	0	0	1 (4.2)	4	0	0	0	0	0	0	0	63	2 (3.2)	0	5 (7.9)
18	TAPVR	2	0	0	0	2	0	0	0	0	0	0	0	0	0	0	0	4	0	0	0
19	PAPVR ± ASD	0	0	0	0	0	0	0	0	2	0	0	0	0	0	0	0	2	0	0	0
20	ASD	0	0	0	0	0	0	0	0	8	0	0	0	11	0	0	0	19	0	0	0
21	Cor triatriatum	0	0	0	0	0	0	0	0	0	0	0	0	0	0	0	0	0	0	0	0
22	AVSD (partial)	1	0	0	0	1	0	0	0	0	0	0	0	0	0	0	0	2	0	0	0
23	AVSD (complete)	32	0	0	1 (3.1)	74	0	0	0	5	0	0	0	0	0	0	0	111	0	0	1 (0.9)
24	+TOF or DORV	2	0	0	0	13	0	0	0	2	0	0	0	0	0	0	0	17	0	0	0
25	+Others	7	2 (28.6)	0	2 (28.6)	1	0	0	0	1	0	0	0	0	0	0	0	9	2 (22.2)	0	2 (22.2)
26	VSD (subarterial)	3	0	0	0	11	0	0	0	0	0	0	0	0	0	0	0	14	0	0	0
27	VSD (perimemb/muscular)	37	0	0	0	116	2 (1.7)	0	2 (1.7)	5	0	0	0	3	0	0	0	161	2 (1.2)	0	2 (1.2)
28	VSD + PS	0	0	0	0	2	0	0	0	0	0	0	0	0	0	0	0	2	0	0	0
29	DCRV ± VSD	0	0	0	0	1	0	0	0	0	0	0	0	1	0	0	0	2	0	0	0
30	Aneurysm of sinus valsalva	1	1 (100.0)	0	1 (100.0)	1	1 (100.0)	0	1 (100.0)	0	0	0	0	0	0	0	0	2	2 (100.0)	0	2 (100.0)
31	TOF	25	1 (4.0)	0	1 (4.0)	126	1 (0.8)	0	1 (0.8)	17	0	0	0	2	0	0	0	170	2 (1.2)	0	2 (1.2)
32	PA + VSD	21	0	0	0	64	0	0	0	18	0	0	0	2	0	0	1 (50.0)	105	0	0	1 (1.0)
33	DORV	35	1 (2.9)	0	2 (5.7)	55	1 (1.8)	0	2 (3.6)	7	0	0	0	1	0	0	0	98	2 (2.0)	0	4 (4.1)
34	TGA (simple)	1	0	0	0	3	0	0	0	0	0	0	0	0	0	0	0	4	0	0	0
35	+VSD	4	0	0	0	2	0	0	0	1	0	0	0	1	0	0	0	8	0	0	0
36	VSD + PS	6	0	0	0	8	0	0	0	0	0	0	0	0	0	0	0	14	0	0	0
37	Corrected TGA	7	0	0	0	15	0	0	0	8	0	0	0	0	0	0	0	30	0	0	0
38	Truncus arteriosus	20	1 (5.0)	0	1 (5.0)	10	1 (10.0)	0	1 (10.0)	0	0	0	0	0	0	0	0	30	2 (6.7)	0	2 (6.7)
39	SV	69	1 (1.4)	0	1 (1.4)	59	2 (3.4)	0	4 (6.8)	16	0	0	0	2	0	0	0	146	3 (2.1)	0	5 (3.4)
40	TA	16	1 (6.3)	0	1 (6.3)	20	0	0	0	5	0	0	0	1	0	0	0	42	1 (2.4)	0	1 (2.4)
41	HLHS	78	1 (1.3)	0	5 (6.4)	9	2 (22.2)	0	2 (22.2)	7	0	0	0	0	0	0	0	94	3 (3.2)	0	7 (7.4)
42	Aortic valve lesion	3	0	0	0	0	0	0	0	2	0	0	0	0	0	0	0	5	0	0	0
43	Mitral valve lesion	0	0	0	0	0	0	0	0	0	0	0	0	0	0	0	0	0	0	0	0
44	Ebstein	2	1 (50.0)	0	1 (50.0)	7	0	0	0	2	0	0	0	0	0	0	0	11	1 (9.1)	0	1 (9.1)
45	Coronary disease	1	0	0	0	1	0	0	0	3	0	0	0	0	0	0	0	5	0	0	0
46	Others	11	0	0	0	18	1 (5.6)	0	1 (5.6)	35	0	0	1 (2.9)	8	0	0	0	72	1 (1.4)	0	2 (2.8)
47	Redo VSD	0	0	0	0	0	0	0	0	1	0	0	0	0	0	0	0	1	0	0	0
48	PS release	0	0	0	0	3	0	0	0	4	0	0	0	2	0	0	0	9	0	0	0
49	RV-PA conduit replace	0	0	0	0	0	0	0	0	0	0	0	0	0	0	0	0	0	0	0	0
50	Others	13	0	0	0	23	0	0	0	24	0	0	0	3	0	0	0	63	0	0	0
Total		1006	20 (2.0)	0	35 (3.5)	947	14 (1.5)	0	21 (2.2)	221	0	0	1 (0.5)	42	0	0	1 (2.4)	2216	34 (1.5)	0 (0.00)	58 (2.6)

Values in parenthesis represent mortality %

*CPB* cardiopulmonary bypass, *PDA* patient ductus arteriosus, *VSD* ventricular septal defect, *DORV* double outlet right ventricle, *AVSD* atrioventricular septal defect, *TGA* transposition of great arteries, *SV* single ventricle, *Interupt. of Ao.* interruption of aorta, *PS* pulmonary stenosis, *PA-IVS* pulmonary atresia with intact ventricular septum, *TAPVR* total anomalous pulmonary venous return, *PAPVR* partial anomalous pulmonary venous return, *ASD* atrial septal defect, *TOF* tetralogy of Fallot, *DCRV* double-chambered right ventricle, *TA* tricuspid atresia, *HLHS* hypoplastic left heart syndrome, *RV-PA* right ventricle-pulmonary artery

**Table 5:** (3) Main procedure

		Neonate				Infant				1–17 years				≧18 years				Total			
		Cases	30-day mortality		Hospital mortality	Cases	30-day mortality		Hospital mortality	Cases	30-day mortality		Hospital mortality	Cases	30-day mortality		Hospital mortality	Cases	30-day mortality		Hospital mortality
			Hospital	After discharge			Hospital	After discharge			Hospital	After discharge			Hospital	After discharge			Hospital	After discharge	
1	SP shunt	182	5 (2.7)	0	11 (6.0)	395	8 (2.0)	0	12 (3.0)	67	0	0	0	5	0	0	1 (20.0)	649	13 (2.0)	0	24 (3.7)
2	PAB	375	11 (2.9)	0	19 (5.1)	282	5 (1.8)	0	9 (3.2)	18	0	0	0	4	0	0	0	679	16 (2.4)	0	28 (4.1)
3	Bidirectional Glenn or hemi-Fontan ± α	15	0	0	1 (6.7)	254	3 (1.2)	0	5 (2.0)	84	1 (1.2)	0	1 (1.2)	3	0	0	0	356	4 (1.1)	0	7 (2.0)
4	Damus–Kaye–Stansel operation	3	1 (33.3)	0	1	50	4	0	6	24	0	0	0	0	0	0	0	77	5 (6.5)	0	7 (9.1)
5	PA reconstruction/repair (including redo)	12	0	0	0	72	4 (5.6)	0	7 (9.7)	107	1 (0.9)	0	2 (1.9)	15	0	0	0	206	5 (2.4)	0	9 (4.4)
6	RVOT reconstruction/repair	22	2 (9.1)	0	2 (9.1)	105	1 (1.0)	0	1 (1.0)	163	1 (0.6)	0	1 (0.6)	22	0	0	0	312	4 (1.3)	0	4 (1.3)
7	Rastelli procedure	5	0	0	0	67	2 (3.0)	0	2 (3.0)	98	0	0	0	10	0	0	0	180	2 (1.1)	0	2 (1.1)
8	Arterial switch procedure	129	5 (3.9)	0	6 (4.7)	27	2 (7.4)	0	2 (7.4)	8	0	0	0	0	0	0	0	164	7 (4.3)	0	8 (4.9)
9	Atrial switch procedure	3	0	0	0	0	0	0	0	1	0	0	0	0	0	0	0	4	0	0	0
10	Double switch procedure	0	0	0	0	2	0	0	0	10	0	0	0	1	0	0	0	13	0	0	0
11	Repair of anomalous origin of CA	1	0	0	0	9	0	0	0	4	0	0	0	6	0	0	0	20	0	0	0
12	Closure of coronary AV fistula	3	0	0	0	1	0	0	0	5	0	0	0	21	1 (4.8)	0	1 (4.8)	30	1 (3.3)	0	1 (3.3)
13	Fontan/TCPC	3	0	0	0	6	0	0	0	417	4 (1.0)	0	5 (1.2)	24	2 (8.3)	0	2 (8.3)	450	6 (1.3)	0	7 (1.6)
14	Norwood procedure	35	5 (14.3)	0	9 (25.7)	70	5 (7.1)	0	11 (15.7)	3	0	0	0	0	0	0	0	108	10 (9.3)	0	20 (18.5)
15	Ventricular septation	0	0	0	0	1	0	0	0	0	0	0	0	1	0	0	0	2	0	0	0
16	Left side AV valve repair (including Redo)	2	0	0	0	51	0	0	0	64	0	0	0	17	0	0	0	134	0	0	0
17	Left side AV valve replace (including Redo)	2	0	0	0	16	1 (6.3)	0	2 (12.5)	40	1 (2.5)	0	1 (2.5)	15	0	0	0	73	2 (2.7)	0	3 (4.1)
18	Right side AV valve repair (including Redo)	8	2 (25.0)	0	2 (25.0)	21	0	0	0	25	0	0	0	23	0	0	1 (4.3)	77	2 (2.6)	0	3 (3.9)
19	Right side AV valve replace (including Redo)	0	0	0	0	6	0	0	1 (16.7)	2	0	0	0	14	0	0	0	22	0	0	1 (4.5)
20	Common AV valve repair (including Redo)	2	1 (50.0)	0	1 (50.0)	19	1 (5.3)	0	2 (10.5)	28	1 (3.6)	0	1 (3.6)	3	0	0	0	52	3 (5.8)	0	4 (7.7)
21	Common AV valve replace (including Redo)	0	0	0	0	2	0	0	0	6	0	0	1 (16.7)	0	0	0	0	8	0	0	1 (12.5)
22	Repair of supra-aortic stenosis	0	0	0	0	1	0	0	0	19	0	0	0	5	0	0	0	25	0	0	0
23	Repair of subaortic stenosis (including Redo)	3	0	0	0	9	0	0	0	29	0	0	0	5	0	0	0	46	0	0	0
24	Aortic valve plasty ± VSD Closure	6	0	0	0	12	0	0	1 (8.3)	21	0	0	0	6	0	0	0	45	0	0	1 (2.2)
25	Aortic valve replacement	0	0	0	0	1	0	0	0	25	1 (4.0)	0	1 (4.0)	26	0	0	0	52	1 (1.9)	0	1 (1.9)
26	AVR with annular enlargement	0	0	0	0	0	0	0	0	11	0	0	0	1	0	0	0	12	0	0	0
27	Aortic root Replace (except Ross)	0	0	0	0	1	0	0	0	4	0	0	0	6	0	0	0	11	0	0	0
28	Ross procedure	0	0	0	0	3	0	0	0	13	0	0	0	0	0	0	0	16	0	0	0
Total		811	32 (3.9)	0 (0.0)	52 (6.4)	1483	36 (2.4)	0	61 (4.1)	1296	10 (0.8)	0	13 (1.0)	233	3 (1.3)	0	5 (2.1)	3823	81 (2.1)	0	131 (3.4)

Values in parenthesis represent mortality %

*SP* systemic-pulmonary, *PAB* pulmonary artery banding, *PA* pulmonary artery, *RVOT* right ventricular outflow tract, *CA* coronary artery, *AV* *fustula* arteriovenous fistula, *TCPC* total cavopulmonary connection, *AV valve* atrioventricular valve, *VSD* ventricular septal defect, *AVR* aortic valve replacement

**Table 6:** **Table 4** Acquired (total, (1) + (2) + (4) + (5) + (6) + (7) + isolated ope. for arrhythmia in (3); 40,039 (1) Valvular heart disease (total; 21,758)

	Valve	Cases	Operation					30-day mortality				Hospital mortality		Redo			
			Mechanical	Bioprosthesis	Ross Procedure	Repair	With CABG	Hospital		After discharge				Cases	30-day mortality		Hospital mortality
								Replace	Repair	Replace	Repair	Replace	Repair		Hospital	After discharge	
Isolated	A	10,379	2040	7994	5	340	2405	221 (2.2)	6 (1.8)	5 (0.05)	0	294 (2.9)	8 (2.4)	397	28 (7.1)	0	36 (9.1)
	M	4793	697	882	0	3214	735	59 (3.7)	25 (0.8)	0	0	86 (5.4)	35 (1.1)	354	13 (3.7)	0	20 (5.6)
	T	306	7	58		241	34	0	2 (0.8)	0	0	2 (3.1)	4 (1.7)	47	0	0	1 (2.1)
	P	8	0	6		2	1	0	0	0	0	0	0	4	0	0	0
A + M	A	1443	392	992	0	59	246	55 (3.8)		0		67 (4.6)		89	7 (7.9)	0	10 (11.2)
	M		285	416	0	742											
A + T	A	445	105	329	0	11	49	10 (2.2)		0		19 (4.3)		47	0	0	2 (4.3)
	T		3	19	2	421											
M + T	M	3369	583	902		1884	304	49 (1.5)		3 (0.1)		85 (2.5)		208	7 (3.4)	1 (0.5)	10 (4.8)
	T		5	66		3298											
A + M + T	A	972	261	685	0	26	103	47 (4.8)		2 (0.2)		67 (6.9)		74	6 (8.1)	0	12 (16.2)
	M		200	380	1	391											
	T		5	17	0	950											
Others		43	12	24	0	20	3	2 (4.7)		0		2 (4.7)		14	0	0	0
Total		21,758	4595	12,770	8	11,599	3880	476 (2.2)		10 (0.05)		669 (3.1)		1234	61 (4.9)	1 (0.1)	91 (7.4)

Number of redo cases is included in total case number of 21,758

Values in parenthesis represent mortality %

*CABG* coronary artery bypass grafting, *A* aortic valve, *M* mitral valve, *T* tricuspid valve, *P* pulmonary valve

**Table 7:** (2) Ischemic heart disease (total, (A) + (B) + (C); 16,560) (*A*) *Isolated CABG* (*total*; (*a*)+(*b*); *15,333*) (a-1) on-pump arrest CABG (total; 3422)

	Primary, elective				Primary, emergency				Redo, elective				Redo, emergency				Arterial graft only	Artery graft + SVG	SVG only	Others
	Cases	30-day mortality		Hospital mortality	Cases	30-day mortality		Hospital mortality	Cases	30-day mortality		Hospital mortality	Cases	30-day mortality		Hospital mortality				
		Hospital	After discharge			Hospital	After discharge			Hospital	After discharge			Hospital	After discharge					
1VD	103	1 (1.0)	0	2 (1.9)	26	1 (3.8)	0	2 (7.7)	6	1 (16.7)	0	1 (16.7)	1	0	0	0	61	22	52	1
2VD	504	1 (0.2)	0	2 (0.4)	57	8 (14.0)	0	8 (14.0)	3	0	0	0	0	0	0	0	84	447	33	0
3VD	1523	13 (0.9)	0	19 (1.2)	167	6 (3.6)	0	8 (4.8)	14	0	0	0	2	0	0	0	66	1592	48	0
LMT	789	11 (1.4)	0	20 (2.5)	220	9 (4.1)	0	12 (5.5)	5	0	0	0	2	0	0	0	102	872	39	3
Total	2919	26 (0.9)	0	43 (1.5)	470	24 (5.1)		30 (6.4)	28	1 (3.6)		1 (3.6)	5	0		0	313	2933	172	4
Kawasaki	8	0	0	0	0	0	0	0	1	0	0	0	0	0	0	0	6	3	0	0
Hemodialysis	177	2 (1.1)	0	4 (2.3)	27	2 (7.4)	0	2 (7.4)	3	0	0	0	1	0	0	0	6	190	12	0

Values in parenthesis represent mortality %

*CABG* coronary artery bypass grafting, *1VD* one-vessel disease, *2VD* two-vessel disease, *3VD* three-vessel disease, *LMT* left main trunk, *SVG* saphenous vein graft, *LMT* includes LMT alone or LMT with other branch diseases

**Table 8:** (a-2) On-pump beating CABG (total; 2121)

	Primary, elective				Primary, emergency				Redo, elective				Redo, emergency				Arterial graft only	Artery graft + SVG	SVG only	Others	Unclear
	Cases	30-day mortality		Hospital mortality	Cases	30-day mortality		Hospital mortality	Cases	30-day mortality		Hospital mortality	Cases	30-day mortality		Hospital mortality					
		Hospital	After discharge			Hospital	After discharge			Hospital	After discharge			Hospital	After discharge						
1VD	42	1 (2.4)	0	2 (4.8)	17	4 (23.5)	0	4 (23.5)	11	0	0	1 (9.1)	5	2 (40.0)	0	2 (40.0)	34	10	30	0	1
2VD	248	1 (0.4)	0	2 (0.8)	84	13 (15.5)	0	18 (21.4)	4	0	0	0	1	0	0	0	58	249	28	1	1
3VD	860	14 (1.6)	0 (0.0)	27 (3.1)	196	18 (9.2)	0	22 (11.2)	12	0	0	0	0	0	0	0	90	921	57	0	0
LMT	434	5 (1.2)	1 (0.2)	5 (1.2)	195	13 (6.7)	0 (0.0)	17 (8.7)	10	0	0	0	2	1 (50.0)	0	1 (50.0)	87	521	31	0	2
Total	1584	21 (1.3)	1 (0.1)	36 (2.3)	492	48 (9.8)		61 (12.4)	37	0		1 (2.7)	8	3 (37.5)		3 (37.5)	269	1701	146	1	0
Kawasaki	1	0	0	0	0	0	0	0	0	0	0	0	0	0	0	0	1	0	0	0	0
Hemodialysis	154	2 (1.3)	0	4 (2.6)	67	5 (7.5)	0	8 (11.9)	3	0	0	0	2	0	0	0	14	193	18	0	1

Values in parenthesis represent mortality %

*CABG* coronary artery bypass grafting, *1VD* one-vessel disease, *2VD* two-vessel disease, *3VD* three-vessel disease, *LMT* left main trunk, *SVG* saphenous vein graft, *LMT* includes LMT alone or LMT with other branch diseases

**Table 9:** (b) off-pump CABG (total; 9790) (The present section also includes cases of planned off-pump CABG in which, during surgery, the change is made to an on-pump CABG or on-pump beating-heart procedure)

	Primary, elective				Primary, emergency				Redo, elective				Redo, emergency				Arterial graft only	Artery graft + SVG	SVG only	Others
	Cases	30-day mortality		Hospital mortality	Cases	30-day mortality		Hospital mortality	Cases	30-day mortality		Hospital mortality	Cases	30-day mortality		Hospital mortality				
		Hospital	After discharge			Hospital	After discharge			Hospital	After discharge			Hospital	After discharge					
1VD	573	2 (0.3)	0	3 (0.5)	73	3 (4.1)	0	8 (11.0)	34	1 (2.9)	0	1 (2.9)	9	0	0	0	570	47	72	0
2VD	1533	13 (0.8)	0	26 (1.7)	150	7 (4.7)	0	10 (6.7)	19	0	0	0	2	0	0	0	625	1024	55	0
3VD	3947	30 (0.8)	0	50 (1.3)	383	13 (3.4)	0	20 (5.2)	12	0	0	1 (8.3)	1	0	0	0	840	3433	70	2
LMT	2468	8 (0.3)	1 (0.0)	19 (0.8)	553	21 (3.8)	0	25 (4.5)	28	0	0	0	5	0	0	2 (40.0)	818	2168	68	0
Total	8521	53 (0.6)	1 (0.0)	98 (1.2)	1159	44 (3.8)		63 (5.4)	93	1 (1.1)		2 (2.2)	17	0		2 (11.8)	2853	6672	265	2
Kawasaki	7	0	0	0	1	0	0	0	0	0	0	0	0	0	0	0	6	1	1	0
Hemodialysis	581	7 (1.2)	0	15 (2.6)	79	7 (8.9)	0	9 (11.4)	7	0	0	0	5	0	0	0	131	511	30	0

Values in parenthesis represent mortality %

*CABG* coronary artery bypass grafting, *1VD* one-vessel disease, *2VD* two-vessel disease, *3VD* three-vessel disease, *LMT* left main trunk, *SVG* saphenous vein graft, *LMT* includes LMT alone or LMT with other branch diseases

**Table 10:** (c) Includes cases of conversion, during surgery, from off-pump CABG to on-pump CABG or on- pump beating-heart CABG (total; 171)

	Primary, elective				Primary, emergency				Redo, elective				Redo, emergency			
	Cases	30-day mortality		Hospital mortality	Cases	30-day mortality		Hospital mortality	Cases	30-day mortality		Hospital mortality	Cases	30-day mortality		Hospital mortality
		Hospital	After discharge			Hospital	After discharge			Hospital	After discharge			Hospital	After discharge	
A conversion to on-pump CABG arrest heart	33	2 (6.1)	0	2 (6.1)	3	0	0	0	0	0	0	0	0	0		0
A conversion to on-pump beating-heart CABG	104	4 (3.8)	0	5 (4.8)	31	4 (12.9)	0	4 (12.9)	0	0	0	0	0	0		0
Total	137	6 (4.4)	0	7 (5.1)	34	4 (11.8)		4 (11.8)	0	0	0	0	0	0	0	0
Hemodialysis	10	1 (10.0)	0	1 (10.0)	3	2 (66.7)		2 (66.7)	0	0	0	0	0	0	0	0

Values in parenthesis represent mortality %

*CABG* coronary artery bypass grafting

**Table 11:** (*B*) *Operation for complications of MI* (*total; 1226*)

	Chronic				Acute				Concomitant operation		
	Cases	30-day mortality		Hospital mortality	Cases	30-day mortality		Hospital mortality			
		Hospital	After discharge			Hospital	After discharge		CABG	MVP	MVR
Infarctectomy or aneurysmectomy	336	10 (3.0)	0	17 (5.1)	29	5 (17.2)	1 (3.4)	7 (24.1)	247	115	19
VSP closure	57	5 (8.8)	1 (1.8)	5 (8.8)	221	64 (29.0)	3 (1.4)	85 (38.5)	76	2	5
Cardiac rupture	21	3 (14.3)	0	4 (19.0)	176	52 (29.5)	1 (0.6)	59 (33.5)	23	1	3
Mitral regurgitation											
1) Papillary muscle rupture	9	0	0	0	43	10 (23.3)	0	12 (27.9)	20	3	36
2) Ischemic	289	11 (3.8)	0	26 (9.0)	30	11 (36.7)	1 (3.3)	13 (43.3)	254	222	55
Others	8	2 (25.0)	0	2 (25.0)	7	1 (14.3)	0	1 (14.3)	6	0	1
Total	720	31 (4.3)	1 (0.1)	54 (7.5)	506	143 (28.3)	6 (1.2)	177 (35.0)	626	343	119

Values in parenthesis represent mortality %

Acute, within 2 weeks from the onset of myocardial infarction

*MI* myocardial infarction, *CABG* coronary artery bypass grafting, *MVP* mitral valve repair, *MVR* mitral valve replacement, *VSP* ventricular septal perforation

**Table 12:** (*C*) *TMLR* (*total; 1*)

	Cases	30-day mortality		Hospital mortality
		Hospital	After discharge	
Isolated	1	0	0	0
With CABG	0	0	0	0
Total	1	0	0	0

*TMLR* transmyocardial laser revascularization

**Table 13:** (3) Operation for arrhythmia (total; 4000)

	Cases	30-day mortality		Hospital mortality	Concomitant operation						
					Isolated	Congenital	Valve	IHD	Others	Multiple combination	
		Hospital	After discharge							2 categories	3 categories
Maze	3763	40 (1.1)	2 (0.05)	59 (1.6)	64	159	3338	479	189	421	28
For WPW	0	0	0	0	0	0	0	0	0	0	0
For ventricular tachyarrythmia	43	1 (2.3)	0	1 (2.3)	3	1	13	23	8	5	0
Others	194	2 (1.0)	0	4 (2.1)	1	19	139	50	24	33	3
Total	4000	43 (1.1)	2 (0.05)	64 (1.6)	68	179	3490	552	221	459	31

Values in parenthesis represent mortality %. Except for 68 isolated cases, all remaining 3932 cases are doubly allocated, one for this subgroup and the other for the subgroup corresponding to the concomitant operations

*WPW* Wolff–Parkinson–White syndrome, *IHD* ischemic heart disease

**Table 14:** (4) Operation for constrictive pericarditis (total; 198)

	CPB (+)				CPB (−)			
	Cases	30-day mortality		Hospital mortality	Cases	30-day mortality		Hospital mortality
		Hospital	After discharge			Hospital	96	
Total	99	5 (5.1)	0	7 (7.1)	99	2 (2.0)	0	4 (4.0)

Values in parenthesis represent mortality %

*CPB* cardiopulmonary bypass

**Table 15:** (5) Cardiac tumor (total; 634)

	Cases	30-day mortality		Hospital mortality	Concomitant operation			
		Hospital	After discharge		AVR	MVR	CABG	Others
Benign tumor	550	5 (0.9)	3 (0.5)	6 (1.1)	12	13	31	61
Cardiac myxoma	404	4 (1.0)	3 (0.7)	4 (1.0)	9	8	21	42
Papillary fibroelastoma	63	0	0	0	3	2	7	13
Rhabdomyoma	1	0	0	0	0	0	0	0
Others	82	1 (1.2)	0	2 (2.4)	0	3	3	3
Malignant tumor	84	3 (3.6)	1 (1.2)	9 (10.7)	0	3	5	11
Primary	52	1 (1.9)	1 (1.9)	6 (11.5)	0	3	3	2
Metastatic	32	2 (6.3)	0	3 (9.4)	0	0	2	9

Values in parenthesis represent mortality %

*AVR* aortic valve replacement, *MVR* mitral valve replacement, *CABG* coronary artery bypass grafting

**Table 16:** (6) HOCM and DCM (total; 240)

	Cases	30-day mortality		Hospital mortality	Concomitant operation			
		Hospital	After discharge		AVR	MVR	MVP	CABG
Myectomy	162	1 (0.6)	0	1 (0.6)	86	27	22	16
Myotomy	4	0	0	1 (25.0)	0	1	1	0
No-resection	38	3 (7.9)	0	5 (13.2)	4	20	14	2
Volume reduction surgery of the left ventricle	36	3 (8.3)	0	5 (13.9)	1	6	19	5
Total	240	7 (2.9)	0	12 (5.0)	91	54	56	23

Values in parenthesis represent mortality %

*HOCM* hypertrophic obstructive cardiomyopathy, *DCM* dilated cardiomyopathy, *AVR* aortic valve replacement, *MVR* mitral valve replacement, *MVP* mitral valve repair, *CABG* coronary artery bypass grafting

**Table 17:** (7) Other open-heart operation (total; 586)

	Cases	30-day mortality		Hospital mortality
		Hospital	After discharge	
Total	586	32 (5.5)	0	45 (7.7)

Values in parenthesis represent mortality %

**Table 18:** **Table 5** Thoracic aortic aneurysm (total; 15,758) (1) Dissection (total; 6787)

Replaced site	Stanford type																								
	Acute								Chronic								Concomitant operation					Redo			
	A				B				A				B												
	Cases	30-day mortality		Hospital mortality	Cases	30-day mortality		Hospital mortality	Cases	30-day mortality		Hospital mortality	Cases	30-day mortality		Hospital mortality	AVP	AVR	MVP	MVR	CABG	Cases	30-day mortality		Hospital mortality
		Hospital	After discharge			Hospital	After discharge			Hospital	After discharge			Hospital	After discharge								Hospital	After discharge	
1. Ascending Ao.	2608	186 (7.1)	2 (0.1)	217 (8.3)	4	0 (0.0)	0	1 (25.0)	186	3 (1.6)	0	6 (3.2)	8	0 (0.0)	0	0 (0.0)	216	111	17	8	132	51	2 (3.9)	0	4 (7.8)
2. Aortic Root	197	34 (17.3)	0	35 (17.8)	0	0	0	0	65	6 (9.2)	0	7 (10.8)	1	0	0	0	37	185	6	1	42	38	5 (13.2)	0	5 (13.2)
3. Ascending Ao. + Arch	1393	108 (7.8)	0	124 (8.9)	32	1 (3.1)	0	2 (6.3)	282	6 (2.1)	0	11 (3.9)	112	3 (2.7)	0	4 (3.6)	106	62	3	4	95	91	4 (4.4)	0	8 (8.8)
4. Arch + descending Ao.	25	1 (4.0)	0	2 (8.0)	6	1 (16.7)	0	2 (33.3)	31	4 (12.9)	0	4 (12.9)	56	4 (7.1)	0	4 (7.1)	0	0	0	0	2	17	6 (35.3)	0	5 (29.4)
5. Aortic Root + Asc. Ao. + Arch	86	12 (14.0)	0	14 (16.3)	2	0	0	0	33	1 (3.0)	0	1 (3.0)	13	0	0	0	22	85	1	0	15	18	0	0	0
6. Descending Ao.	13	3 (23.1)	0	3 (23.1)	37	6 (16.2)	0	5 (13.5)	84	2 (2.4)	0	3 (3.6)	270	12 (4.4)	0	17 (6.3)	0	0	0	0	4	41	1 (2.4)	0	3 (7.3)
7. Thoracoabdominal Ao.	1	0	0	0	11	1 (9.1)	0	3 (27.3)	29	4 (13.8)	0	4 (13.8)	145	9 (6.2)	0	12 (8.3)	0	0	0	0	1	47	5 (10.6)	0	6 (12.8)
8. Extra-anatomical bypass	14	2	0	2 (14.3)	24	2 (8.3)	0	3 (12.5)	2	0	0	0 (0.0)	3	0	0	0	0	0	0	0	0	3	1 (33.3)	0	1 (33.3)
9. Stent graft*^a^	107	9 (8.4)	0	7 (6.5)	181	17 (9.4)	0	21 (11.6)	139	0	0	1 (0.7)	587	6 (1.0)	1	12 (2.0)	6	2	0	0	3	70	2 (2.9)	0	3 (4.3)
1) TEVARl*^b^	48	7 (14.6)	0	7 (14.6)	179	16 (8.9)	0	20 (11.2)	119	0	0	1 (0.8)	556	6 (1.1)	1	12 (2.2)	6	2	0	0	3	70	2 (2.9)	0	3 (4.3)
2) Open stent	59	2 (3.4)	0	0	2	1 (50.0)	0	1 (50.0)	20	0	0	1 (5.0)	31	0	0	12 (38.7)	5	1	0	0	1	68	2 (2.9)	0	3 (4.4)
a) With total arch*^c^	59	2 (3.4)	0	0	2	1 (50.0)	0	1 (50.0)	20	0	0	0	16	0	0	0	1	1	0	0	2	1	0	0	0
b) Without total arch*^d^	0	0	0	0	0	0	0	0	0	0	0	0	15	0	0	0	0	0	0	0	0	1	0	0	0
3) Unspecified	0	0	0	0	0	0	0	0	0	0	0	0	0	0	0	0	0	0	0	0	0	0	0	0	0
Total	4444	355 (8.0)	2 (0.05)	404 (9.1)	297	28 (9.4)	0	37 (12.5)	851	26 (3.1)	0 (0.0)	37 (4.3)	1195	34 (2.8)	1	49 (4.1)	387	445	27	13	294	376	26 (6.9)	0	35 (9.3)

Values in parenthesis represent mortality %

*Ao* aorta, *AVP* aortic valve repair, *AVR* aortic valve replacement, *MVP* mitral valve repair, *MVR* mitral valve replacement, *CABG* coronary artery bypass grafting, *TEVAR* thoracic endovascular aortic (aneurysm) repair

Acute, within 2 weeks from the onset

*a = *b + *c + *d + unspecified

**Table 19:** (2) Non-dissection (total; 8971)

Replaced site	Unruptured				Ruptured				Concomitant operation					Redo				CPB (−)			
	Cases	30-day mortality		Hospital mortality	Cases	30-day mortality		Hospital mortality	AVP	AVR	MVP	MVR	CABG	Cases	30-day mortality		Hospital mortality	Cases	30-day mortality		Hospital mortality
		Hospital	After discharge			Hospital	After discharge								Hospital	After discharge			Hospital	After discharge	
1. Ascending Ao.	1201	25 (2.1)	0	32 (2.7)	62	7 (12.9)	0	9 (14.5)	40	820	67	39	147	95	7 (7.4)	0	10 (10.5)	2	0	0	0
2. Aortic Root	928	16 (1.7)	1 (0.1)	24 (2.6)	31	8 (30.8)	0	8 (25.8)	232	631	54	24	95	145	13 (9.0)	0	14 (9.7)	1	0	0	0
3. Ascending Ao. + Arch	2151	44 (2.0)	0	72 (3.3)	173	25 (14.8)	0	37 (21.4)	33	201	28	8	327	105	8 (7.6)	0	10 (9.5)	6	0	0	0
4. Arch + descending Ao.	104	6 (5.8)	0	9 (8.7)	23	3 (34.3)	0	5 (21.7)	0	2	0	1	7	11	3 (27.3)	0	4 (36.4)	8	0	0	0
5. Aortic root + Asc. Ao. + Arch	109	1 (0.9)	0	2 (1.8)	5	2 (50.0)	0	2 (40.0)	24	80	5	1	6	20	3 (15.0)	0	3 (15.0)	2	0	0	0
6. Descending Ao.	343	12 (3.5)	0	18 (5.2)	84	16 (19.7)	0	18 (21.4)	0	0	0	0	4	33	3 (9.1)	0	6 (18.2)	26	2 (7.7)	0	2 (7.7)
7. Thoracoabdominal Ao.	372	17 (4.6)	1 (0.3)	28 (7.5)	52	9 (24.3)	0	15 (28.8)	0	0	0	0	1	42	1 (2.4)	0	3 (7.1)	11	0	0	0
8. Extra-anatomical bypass	35	0	0	1 (2.9)	2	0 (0.0)	0	0	1	0	0	0	1	1	0	0	0	2	0 (0.0)	0	0
9. Stent graft*^a^	2928	43 (1.5)	1 (0.03)	73 (2.5)	368	39 (12.9)	3 (0.8)	55 (14.9)	7	7	1	0	25	122	6 (4.9)	1 (0.8)	7 (5.7)	1079	19 (1.8)	3 (0.3)	28 (2.6)
1) TEVAR*^b^	2774	37 (1.3)	1 (0.04)	66 (2.4)	358	38 (13.7)	3 (0.8)	53 (14.8)	3	1	0	0	5	118	5 (4.2)	1 (0.8)	6 (5.1)	1059	19 (1.8)	3 (0.3)	28 (2.6)
2) Open stent	154	6 (3.9)	0	7 (4.5)	10	1 (10.0)	0	2 (20.0)	4	6	1	0	20	4	1 (25.0)	0	1 (25.0)	20	0	0	0
a) With total arch*^c^	42	0	0	0	0	0	0	0	0	1	0	0	4	2	0	0	0	20	0	0	0
b) Without total arch*^d^	112	6 (5.4)	0	7 (6.3)	10	1 (10.0)	0	2 (20.0)	4	5	1	0	16	2	1 (50.0)	0	1 (50.0)	0	0	0	0
3) Unspecified	0	0		0	0	0	0	0	0	0	0	0	0	0	0	0	0	0	0	0	0
Total	8171	164 (2.0)	3 (0.04)	259 (3.2)	800	109 (13.6)	3 (0.4)	149 (22.2)	337	1741	155	73	613	574	44 (7.7)	1 (0.2)	57 (9.9)	1137	21 (3.0)	3 (0.3)	30 (2.6)

Values in parenthesis represent mortality %

*Ao* aorta, *AVP* aortic valve repair, *AVR* aortic valve replacement, *MVP* mitral valve repair, *MVR* mitral valve replacement, *CABG* coronary artery bypass grafting, *TEVAR* thoracic endovascular aortic (aneurysm) repair

*a = *b + *c + *d + unspecified

**Table 20:** **Table 6** Pulmonary thromboembolism (total; 176)

	Cases	30-day mortality		Hospital mortality
		Hospital	After discharge	
Acute	114	16 (14.0)	0	17 (14.9)
Chronic	62	3 (4.8)	0	4 (6.5)
Total	176	19 (10.8)	0	21 (11.9)

Values in parenthesis represent mortality %

**Table 21:** **Table 7** Assisted circulation (total; 1713)

Sites	VAD									Heart–lung assist					
	Device			Results						Method		Results			
	Centrifugal	VAS	Others	Not weaned			Weaned			PCPS	Others	Not weaned		Weaned	
				On going	Death	Transplant	Alive	Deaths	Transplant			Deaths	Transplant	Deaths	Alive
Post cardiotomy															
Left	38	4	7	8	30 (61.2)	0	3	8 (19.0)	0						
Right	0	0	0	0	0	0	0	0	0						
Biventricle															
Left	1	3	0	0	3 (75.0)	0	1	0	0	499	69	274 (54.9)	0	85 (17.0)	209
Right	4	0	0												
Congestive heart failure															
Left	50	41	92	112	38 (20.8)	7	16	9 (9.9)	0						
Right	2	1	0	1	0	0	2	0	0						
Biventricle															
Left	5	22	3	5	14 (46.7)	0	8	3 (10.3)	0	685	29	360 (52.6)	0	105 (15.3)	249
Right	18	11	1												
Respiratory failure										106	22	46 (43.4)	0	16 (15.1)	66
Total	118	82	103	126	85 (28.1)	7	30	20 (6.6)	0	1290	120	680 (52.7)	2	206 (16.0)	524

Values in parenthesis represent mortality %

*VAD* ventricular assist devise, *VAS* ventricular assist system, *PCPS* percutaneous cardiopulmonary support

**Table 22:** **Table 8** Heart transplantation (total; 37)

	Cases	30-day mortality		Hospital mortality
		Hospital	After discharge	
Heart transplantation	36	0	0	0
Heart and lung transplantation	1	0	0	0
Total	37	0	0	0

Values in parenthesis represent mortality %

**Table 23:** **Table 9** Pacemaker + ICD (total; 4202)

	Pacemaker			ICD	
	V	A-V	CRT	CRTD	ICD
Initial	508	1836	78	168	210
Exchange	455	901	30	90	184
Unclear	0	0	0	0	0
Total	963	2737	108	258	394

*ICD* implantable cardioverter-defibrillator, *CRTD* cardiac resynchronization therapy devise with incorporated ICD devise

In 2013, 7150 open-heart operations for congenital heart disease were performed with overall hospital mortality of 2.2 % (Table 3). The number of operations for congenital heart disease was quite steady throughout these 10 years (maximum 7386 cases in 2006), while overall hospital mortality decreased gradually from that of 3.7 % in 2003. In detail, the most common disease was atrial septal defect (1321 cases); however, its number deceased to 71.7 % of that in 2003, which might be due to the recent development of catheter closure of atrial septal defect in Japan. Hospital mortality for complex congenital heart disease improved dramatically in the last 10 years such as interrupted aortic arch with ventricular septal defect (6.7 % in 2003 to 4.9 % in 2013), complete atrio-septal defect (5.7–0.6 %), tetralogy of Fallot (2.6–1.4 %), transposition of the great arteries with and without ventricular septal defect (10.5–5.2 % and 7.5–3.6 %, respectively), single ventricle (7.1–5.7 %), and hypoplastic left heart syndrome (27.2–9.1 %). Right heart bypass surgery is now commonly performed (356 bidirectional Glenn procedures excluding 77 Damus–Kaye–Stansel procedures and 450 Fontan type procedures including total cavo-pulmonary connection) with acceptable hospital mortality (2.0 and 1.6 %). Norwood type I procedure was performed in 108 cases with relatively low hospital mortality rate of 18.5 %.

As previously mentioned, the number of operations for valvular heart disease increased by 83.4 % in the last 10 years, and the hospital mortality associated with primary single valve placement was 2.2 and 3.7 % for the aortic and the mitral position, while that for primary mitral valve repair was 0.8 % (Table 4 (1)). However, hospital mortality rate for redo valve surgery were still high, and was 9.1 and 5.6 % for aortic and mitral procedure, respectively. Finally, overall hospital mortality did not show significant improvement during the last 10 years (3.7 % in 2003 and 3.1 % in 2013), which might be partially due to the recent progression of age of the patients. Repair of the valve became popular procedure (436 cases in the aortic, 6231 cases in the mitral, and 4910 cases in the tricuspid), and mitral valve repair constituted 28.6 % of all valvular heart disease operation and 55.5 % of all mitral valve procedure (10,577 procedures), which are similar to those of the last 5 years and increased compared with those of 2003 (21.3 and 38.7 %, respectively). Aortic and mitral valve replacement with bioprosthesis were performed in 10,000 cases and 2580 cases, respectively, with the number consistently increasing in the aortic position. The ratio of prostheses changed dramatically during the last 10 years and the usage of bioprosthesis is 78.1 % at the aortic position (38.2 % in 2003) and 41.9 % at the mitral position (23.4 % in 2003). CABG as a concomitant procedure performed in 17.8 % of operations for all valvular heart disease (12.7 % in 2003).

Isolated CABG was performed in 15,333 cases which were only 72.9 % of that of 10 years ago (2003) (Table 4 (2)). Among these 15,333 cases, off-pump CABG was intended in 9790 cases (63.8 %) with a success rate of 98.3 %; so final success rate of off-pump CABG was 62.7 %. The percentage of intended off-pump CABG was 55.2 % in 2003, and was increased to 60.3 % in 2004, then was kept over 60 % until now. In 15,333 isolated CABG patients, 96.1 % of them received at least one arterial graft, while, all arterial graft CABG was performed in only 23.4 % of them.

The operative and hospital mortality rates associated with primary elective CABG procedures in 13,024 cases were 1.0 and 1.7 %, respectively. Similar data analysis of CABG including primary/redo and elective/emergency data was begun in 2003, and the operative and hospital mortality rates associated with primary elective CABG procedures in 2003 were 1.0 and 1.5 %, respectively; so operative results of primary CABG has been stable. However, hospital mortality of primary emergency CABG in 2121 cases was 5.5 %, which has been improved compared with 9.7 % of hospital mortality rate in 2003. In comparison with data in 2003, the results of conversion improved both conversion rate (3.1–1.7 %) and hospital mortality (8.5–6.4 %).

A total of 1226 patients underwent surgery for complications of myocardial infarction, including 414 operations for a left ventricular aneurysm or ventricular septal perforation or cardiac rupture and 298 operations for ischemic mitral regurgitation.

Operations for arrhythmia were performed mainly as a concomitant procedure in 4000 cases with satisfactory mortality (1.6 % hospital mortality) including 3763 MAZE procedures. MAZE procedure has become quite popular procedure when compared with that in 2003 (1472 cases).

Operations for thoracic aortic dissection were performed in 6787 cases (Table 5). For 4444 Stanford type A acute aortic dissections, hospital mortality was 9.1 %, which was slightly improved compared to that in 2012 (10.6 %) and better than that in 2003 (14.5 %). Operations for a non-dissected thoracic aneurysm were carried out in 8171 cases, with overall hospital mortality of 4.5 %, which was better than that in 2012 (5.4 %). The hospital mortality associated with un-ruptured aneurysm was 2.2 %, and that of ruptured aneurysm was 22.2 %, which remains markedly high.

The number of stent graft procedures remarkably increased recently. A total of 1014 patients with aortic dissection underwent stent graft placement: thoracic endovascular aortic repair (TEVAR) in 902 cases and open stent grafting in 112 cases. The number of TEVAR for type B chronic aortic dissections increased from 77 cases in 2003 to 556 cases in 2013. The hospital mortality rates associated with TEVAR for type B aortic dissection were 11.2 % in acute cases and 2.2 % for chronic cases, respectively.

A total of 3296 patients with non-dissected aortic aneurysm underwent stent graft placement with a dramatic increase compared with that in 2003 (399 cases); TEVAR in 3132 cases (42 % increase compared with that in 2012) and open stent grafting in 164 cases (27.4 % decrease compared with that in 2012). The hospital mortality rates for TEVAR were 2.4 and 14.8 % for non-ruptured and ruptured aneurysm, respectively.

In summary, the total cardiovascular operations increased during 2013 by 3525 cases. With steadily improving results in almost all categories compared with those in 2012.

### (B) General thoracic surgery

**Fig. 2:**
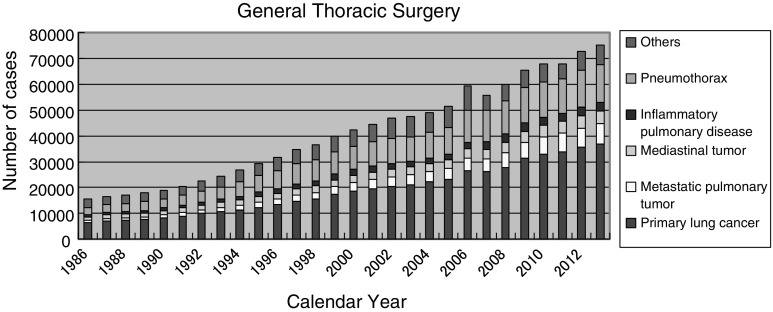
General thoracic surgery

The total number of operations reported in 2013 in general thoracic surgery has reached 75,306, which means increase of 2559 cases compared with the number of operations in 2012 (Fig. 2 Table 10).

**Table 24:** **Table 10** Total entry cases of general thoracic surgery during 2013

	Cases	%
Benign pulmonary tumor	948	1.3
Primary lung cancer	37,008	49.1
Other primary malignant pulmonary tumor	362	0.5
Metastatic pulmonary tumor	7829	10.4
Tracheal tumor	85	0.1
Mesothelioma	439	0.6
Chest wall tumor	692	0.9
Mediastinal tumor	4780	6.3
Thymectomy for MG without thymoma	253	0.3
Inflammatory pulmonary disease	3445	4.6
Empyema	2368	3.1
Bullous disease excluding pneumothorax	566	0.8
Pneumothorax	14,612	19.4
Chest wall deformity	403	0.5
Diapharagmatic hernia including traumatic	101	0.1
Chest trauma excluding diaphragmatic hernia	434	0.6
Lung transplantation	61	0.1
Others	920	1.2
Total	75,306	100.0

Figure 2 shows the development of thoracic surgery in Japan over 27 years. Data for individual categories are summarized in table through 10 to 34. The number of operations for primary lung cancer in 2013 was 37,008, showing the steady increase (31,301, 2009; 32,801, 2010; 33,878, 2011; 35,667, 2012). Surgery for lung cancer consists of 49.1 % of all the general thoracic surgery. Among lung cancer subtypes, adenocarcinoma comprises an overwhelming percentage of 69.1 % of the total lung cancer surgery, followed by squamous cell carcinoma of 19.9 %. Limited resection by wedge resection or segmentectomy was performed in 8771 lung cancer patients, which is 23.7 % of the entire cases. Lobectomy was performed in 27,469 patients, which is 74.2 % of the entire cases. Sleeve lobectomy was done in 449 patients. Pneumonectomy was done in 559 patients which is only 1.5 % of the entire cases. VATS (video assisted thoracic surgery) procedure is performed in 70.8 % among the total lung cancer surgeries. VATS procedure was adopted in 4270 patients (86.2 %) in wedge resection, 2800 patients (73.4 %) in segmentectomy, 18,925 patients (68.9 %) in lobectomy, and 82 patients (14.7 %) in pneumonectomy. There were 123 patients who died within 30 days after lung cancer surgery (30-day mortality rate; 0.33 %), and 224 patients died without discharge (hospital mortality rate; 0.60 %). 30-day mortality rate in regard to procedures is 0.21 % in segmentectomy, 0.34 % in lobectomy, and 1.97 % in pneumonectomy (Table 12).

**Table 25:** **Table 11** 1. Benign pulmonary tumor

	Cases	30 day mortality		Hospital mortality	By VATS
		Hospital	After discharge		
Hamartoma	475	0	0	0	409
Sclerosing hemangioma	95	0	0	0	77
Papilloma	17	0	0	0	13
Mucous gland adenoma bronchial	7	0	0	0	5
Fibroma	60	0	0	0	55
Lipoma	12	0	0	0	9
Neurogenic tumor	9	0	0	0	6
Clear cell tumor	5	0	0	0	4
Leiomyoma	12	0	0	0	9
Chondroma	3	0	0	0	2
Inflamatory myofibroblastic tumor	1	0	0	0	1
Pseudolymphoma	30	0	0	0	21
Histiocytosis	9	0	0	0	9
Teratoma	3	0	0	0	1
Others	210	1 (0.5)	0	1 (0.5)	168
Total	948	1 (0.1)	0	1 (0.1)	789

Values in parenthesis represent mortality %

**Table 26:** **Table 12** 2. Primary malignant pulmonary tumor

	Cases	30 day mortality		Hospital mortality	By VATS
		Hospital	After discharge		
2. Primary malignant pulmonary tumor	37,370	113 (0.3)	11 (0.03)	227 (0.6)	
Lung cancer	37,008	112 (0.3)	11 (0.03)	225 (0.6)	26,213
Adenocarcinoma	25,555	54 (0.2)	4 (0.02)	97 (0.4)	
Squamous cell carcinoma	7365	41 (0.6)	6 (0.08)	89 (1.2)	
Large cell carcinoma	908	6 (0.7)	1	10 (1.1)	
(*LCNEC*)	*517*	*3* (*0.6*)	*1*	*6* (*1.2*)	
Small cell carcinoma	577	3 (0.5)	0	5 (0.9)	
Adenosquamous carcinoma	586	1 (0.2)	0	7 (1.2)	
Carcinoma with pleomorphic, sarcomatoid or sarcomatous elements	496	5 (1.0)	0	11 (2.2)	
Carcinoid	248	0	0	0	
Carcinomas of salivary-gland type	45	0	0	0	
Unclassified	65	0	0	0	
Multiple lung cancer	1015	1 (0.1)	0	3 (0.3)	
Others	148	1 (0.7)	0	3 (2.0)	
Wedge resection	4954	8 (0.2)	1	13 (0.3)	4270
Segmental excision	3817	8 (0.2)	0	15 (0.4)	2800
(*Sleeve segmental excision*)	*13*	*0*	*0*	*0*	*3*
Lobectomy	27,469	84 (0.3)	9 (0.03)	173 (0.6)	18,925
(*Sleeve lobectomy*)	*449*	*4* (*0.9*)	*1* (*0.2*)	*5* (*1.1*)	*54*
Pneumonectomy	559	10 (1.8)	1	20 (3.6)	82
(*Sleeve pneumonectomy*)	*10*	*0*	*0*	*0*	*1*
Other bronchoplasty	6	0	0	0	0
Pleuropneumonectomy	6	0	0	0	0
Others	215	2 (0.9)	0	3 (1.4)	136
Sarcoma	28	0	0	1 (3.6)	
AAH	165	0	0	0	
Others	169	0	0	1 (0.6)	

Values in parenthesis represent mortality %

**Table 27:** **Table 13** Details of lung cancer operation

	Cases
c-Stage (TNM)	
Ia	21,482
Ib	7419
IIa	2939
IIb	1814
IIIa	2587
IIIb	233
IV	400
NA	136
Total	37,010
Sex	
Male	22,996
Female	14,007
NA	7
Total	37,010
Cause of death	
Cardiovascular	17
Pneumonia	43
Pyothorax	8
Bronchopleural fistula	15
Respiratory failure	18
Pulmonary embolism	2
Interstitial pneumonia	84
Brain infarction or bleeding	8
Others	37
Unknown	5
Total	237
p-Stage	
0 (pCR)	222
Ia	18,516
Ib	7777
IIa	3057
IIb	2027
IIIa	3905
IIIb	263
IV	979
NA	264
Total	37,010
Age	
<20	5
20–29	26
30–39	249
40–49	1030
50–59	3699
60–69	12,589
70–79	14,981
80–89	4334
≥90	88
NA	9
Total	37,010

**Table 28:** **Table 14** 3. Metastatic pulmonary tumor

	Cases	30 day mortality		Hospital mortality	By VATS
		Hospital	After discharge		
3. Metastatic pulmonary tumor	7829	7 (0.1)	1 (0.01)	16 (0.2)	6323
Colorectal	3898	4	0	8 (0.2)	3203
Hepatobiliary/pancreatic	375	0	0	1 (0.3)	313
Uterine	391	0	0	0	322
Mammary	456	0	0	1 (0.2)	381
Ovarian	72	0	0	0	59
Testicular	55	0	0	0	41
Renal	608	1 (0.2)	0	1 (0.2)	515
Skeletal	163	0	0	0	113
Soft tissue	280	0	0	0	209
Otorhinolaryngological	424	0	0	0	324
Pulmonary	382	1 (0.3)	1 (0.3)	2 (0.5)	248
Others	725	1 (0.1)	0	3 (0.4)	595

Values in parenthesis represent mortality %

**Table 29:** **Table 15** 4. Tracheal tumor

	Cases	30 day mortality		Hospital mortality
		Hospital	After discharge	
4. Tracheal tumor	85	1 (1.2)	0	1 (1.2)
(A) Primary malignant tumor (histological classification)				
Squamous cell carcinoma	9	0	0	0
Adenoid cystic carcinoma	15	0	0	0
Mucoepidermoid carcinoma	1	0	0	0
Others	4	0	0	0
Total	29	0	0	0
(B) Metastatic/invasive malignant tumor e.g. invasion of thyroid cancer	35	0	0	0
(C) Benign tracheal tumor (histological classification)				
Papilloma	1	0	0	0
Adenoma	0	0	0	0
Neurofibroma	2	0	0	0
Chondroma	1	0	0	0
Leiomyoma	0	0	0	0
Others	17	1 (5.9)	0	1 (5.9)
Histology unknown	0	0	0	0
Total	21	1 (4.8)	0	1 (4.8)
Operation				
Sleeve resection with reconstruction	27	0	0	0
Wedge with simple closure	12	0	0	0
Wedge with patch closure	0	0	0	0
Total laryngectomy with tracheostomy	5	0	0	0
Others	41	1 (2.4)	0	1 (2.4)
Unknown	0	0	0	0
Total	85	1 (1.2)	0	1 (1.2)

Values in parenthesis represent mortality %

**Table 30:** **Table 16** 5. Tumor of pleural origin

	Cases	30 day mortality		Hospital mortality
		Hospital	After discharge	
Histological classification				
Solitary fibrous tumor	147	0	0	1 (0.7)
Diffuse malignant pleural mesothelioma	218	4 (1.8)	0	11 (5.0)
Localized malignant pleural mesothelioma	18	0	0	0
Others	56	0	0	1 (1.8)
Total	439	4 (0.9)	0	13 (3.0)
Operative procedure				
Extrapleural pneumonectomy	119	4 (3.4)	0	10 (8.4)
Total pleurectomy	42	0	0	1 (2.4)
Total parietal pleurectomy	0	0	0	0
Partial pleurectomy	0	0	0	0
Exploratory thoracotomy	0	0	0	0
Others	57	0	0	0
Total	218	4 (1.8)	0	11 (5.0)

Values in parenthesis represent mortality %

**Table 31:** **Table 17** 6. Chest wall tumor

	Cases	30 day mortality		Hospital mortality	By VATS
		Hospital	After discharge		
Primary malignant tumor	133	0	0	0	20
Metastatic malignant tumor	213	0	0	0	41
Benign tumor	346	0	0	0	211
Total	692	0	0	0	272

**Table 32:** **Table 18** 7. Mediastinal tumor

	Cases	30 day mortality		Hospital mortality	By VATS
		Hospital	After discharge		
7. Mediastinal tumor	4780	5 (0.1)	0	9 (0.2)	2624
Thymoma*	1904	2 (0.1)	0	4 (0.2)	765
Thymic cancer	279	1 (0.4)	0	1 (0.4)	69
Thymus carcinoid	47	0	0	0	9
Germ cell tumor	243	0	0	1 (0.4)	87
*Benign*	*161*	*0*	*0*	*0*	*77*
*Malignant*	*82*	*0*	*0*	*1* (*1.2*)	*10*
Neurogenic tumor	513	0	0	0	434
Congenital cyst	974	0	0	0	828
Goiter	85	0	0	0	21
Lymphatic tumor	192	0	0	1 (0.5)	101
Excision of pleural recurrence of thymoma	87	2 (2.3)	0	0	47
Others	456	0	0	2 (0.4)	263

Values in parenthesis represent mortality %

* Includes those with myasthenia gravis

**Table 33:** **Table 19** 8. Thymectomy for myasthenia gravis

	Cases	30 day mortality		Hospital mortality	By VATS
		Hospital	After discharge		
8. Thymectomy for myasthenia gravis	524	0	0	1 (0.2)	176
With thymoma	271	0	0	1 (0.4)	85

Values in parenthesis represent mortality %

**Table 34:** **Table 20** 9. Operation for non-neoplasmic disease (A) Inflammatory pulmonary disease

	Cases	30 day mortality		Hospital mortality	
		Hospital	After discharge		
9. Operation for non-neoplasmic disease	22,848	89 (0.4)	7 (0.03)	160 (0.7)	

	Cases	30 day mortality		Hospital mortality	By VATS
		Hospital	After discharge		
(A) Inflammatory pulmonary disease	3445	4 (0.1)	2 (0.1)	11 (0.3)	2682
Tuberculous infection	99	1 (1.0)	0	1 (1.0)	70
Mycobacterial infection	576	0	0	0	444
Fungal infection	447	3 (0.7)	1 (0.2)	6 (1.3)	260
Bronchiectasis	107	0	0	1 (0.9)	65
Tubeculous nodule	268	0	0	0	227
Inflammatory pseudo tumor	1338	0	0	2 (0.1)	1129
Interpulmonary lymph node	158	0	0	0	147
Others	452	0	1 (0.2)	1 (0.2)	340

Values in parenthesis represent mortality %

**Table 35:** **Table 21** 9. Operation for non-neoplasmic disease (B) Empyema

	Cases	30 day mortality		Hospital mortality	By VATS
		Hospital	After discharge		
Acute empyema	1827	25 (1.4)	0	49 (2.7)	1222
With fistula	403	16 (4.0)	0	28 (6.9)	126
Without fistula	1409	9 (0.6)	0	20 (1.4)	1085
Unknown	15	0	0	1 (6.7)	11
Chronic empyema	541	7 (1.3)	1 (0.2)	21 (3.9)	148
With fistula	287	4 (1.4)	1 (0.3)	13 (4.5)	38
Without fistula	247	3 (1.2)	0	8 (3.2)	104
Unknown	7	0	0	0	6
Total	2368	32 (1.4)	1 (0.04)	70 (3.0)	1370

Values in parenthesis represent mortality %

**Table 36:** **Table 22** 9. Operation for non-neoplasmic disease (C) Descending necrotizing mediastinitis

	Cases	30 day mortality		Hospital mortality	By VATS
		Hospital	After discharge		
(C) Descending necrotizing mediastinitis	98	1 (1.0)	0	1 (1.0)	59

Values in parenthesis represent mortality %

**Table 37:** **Table 23** 9. Operation for non-neoplasmic disease (D) Bullous disease

	Cases	30 day mortality		Hospital mortality	By VATS
		Hospital	After discharge		
(D) Bullous disease	566	2 (0.4)	0	2 (0.4)	447
Emphysematous bulla	433	1 (0.2)	0	1 (0.2)	356
Bronchogenic cyst	57	0	0	0	48
Emphysema with LVRS	24	0	0	0	20
Others	52	1 (1.9)	0	1	23

Values in parenthesis represent mortality %

*LVRS* lung volume reduction surgery

**Table 38:** **Table 24** 9. Operation for non-neoplasmic disease (E) Pneumothorax

	Cases	30 day mortality		Hospital mortality	By VATS
		Hospital	After discharge		
(E) Pneumothorax	14,612	29 (0.2)	2 (0.01)	49 (0.3)	13,961
*Spontaneous pneumothorax*					
Operative procedure					
Bullectomy	3049	0	0	0	2912
Bullectomy with additional procedure	8394	2 (0.02)	1 (0.01)	3 (0.04)	8271
Coverage with artificial material	8008	2 (0.02)	1 (0.01)	3 (0.04)	7894
Parietal pleurectomy	45	0	0	0	45
Coverage and parietal pleurectomy	46	0	0	0	43
Others	295	0	0	0	289
Others	422	1 (0.2)	0	1 (0.2)	377
Unknown	15	0	0	0	15
Total	11,880	3 (0.03)	1 (0.01)	4 (0.03)	11,575
*Secondary pneumothorax*					
Associated disease					
COPD	2151	15 (0.7)	1 (0.05)	22 (1.0)	1899
Tumorous disease	79	4 (5.1)	0	8 (10.1)	66
Catamenial	150	0	0	0	146
LAM	31	0	0	0	30
Others (excluding pneumothorax by trauma)	308	7 (2.3)	0	15 (4.9)	237
Unknown	0				30
Operative procedure					
Bullectomy	370	1 (0.3)	0	5 (1.4)	321
Bullectomy with additional procedure	2018	16 (0.8)	0	27 (1.3)	1796
Coverage with artificial material	1853	13 (0.7)	0	24 (1.3)	1659
Parietal pleurectomy	15	0	0	0	12
Coverage and parietal pleurectomy	21	2 (9.5)	0	2 (9.5)	15
Others	129	1 (0.8)	0	1 (0.8)	110
Others	340	9 (2.6)	1 (0.3)	13 (3.8)	268
Unknown	4	0	0	0	1
Total	2732	26 (1.0)	1 (0.04)	45 (1.6)	2386

Values in parenthesis represent mortality %

**Table 39:** **Table 25** 9. Operation for non-neoplasmic disease (F) Chest wall deformity

	Cases	30 day mortality		Hospital mortality
		Hospital	After discharge	
(F) Chest wall deformity	403	0	0	0
Funnel chest	383	0	0	0
Others	20	0	0	0

**Table 40:** **Table 26** 9. Operation for non-neoplasmic disease (G) Diaphragmatic hernia

	Cases	30 day mortality		Hospital mortality	By VATS
		Hospital	After discharge		
(G) Diaphragmatic hernia	101	2 (2.0)	0	3 (3.0)	33
Congenital	43	2 (4.7)	0	3 (7.0)	11
Traumatic	34	0	0	0	10
Others	24	0	0	0	12

Values in parenthesis represent mortality %

**Table 41:** **Table 27** 9. Operation for non-neoplasmic disease (H) Chest trauma

	Cases	30 day mortality		Hospital mortality	By VATS
		Hospital	After discharge		
(H) Chest trauma	434	16 (3.7)	1 (0.2)	16 (3.7)	171

Values in parenthesis represent mortality %

**Table 42:** **Table 28** 9. Operation for non-neoplasmic disease (I) Other respiratory surgery

	Cases	30 day mortality		Hospital mortality	By VATS
		Hospital	After discharge		
(I) Other respiratory surgery	822	3 (0.4)	1 (0.1)	8 (1.0)	500
Arteriovenous malformation	103	1 (1.0)	0	1 (1.0)	94
Pulmonary sequestration	117	0	0	1 (0.9)	84
Others	602	2 (0.3)	1 (0.2)	6 (1.0)	322

Values in parenthesis represent mortality %

**Table 43:** **Table 29** 10. Lung transplantation

	Cases	30 day mortality		Hospital mortality
		Hospital	After discharge	
Single lung transplantation from brain dead donor	17	0	0	0
Bilateral lung transplantation from brain dead donor	24	0	0	1 (4.2)
Lung transplantation from living donor	20	0	0	1 (5.0)
Total of lung transplantation	61	0	0	2 (3.3)
Donor of living donor lung transplantation	31	0	0	0

Values in parenthesis represent mortality %

**Table 44:** **Table 30** 11. Video-assisted thoracic surgery

	Cases	30 day mortality		Hospital mortality
		Hospital	After discharge	
11. Video-assisted thoracic surgery	58,466	84 (0.1)	9 (0.02)	135 (0.2)

Values in parenthesis represent mortality %

(Including Thoracic sympathectomy 160)

**Table 45:** **Table 31** 12. Tracheobronchoplasty

	Cases	30 day mortality		Hospital mortality
		Hospital	After discharge	
12. Tracheobronchoplasty	564	2 (1.2)	0	4 (0.7)
Trachea	102	1 (1.0)	0	2 (2.0)
Sleeve resection with reconstruction	53	0	0	0
Wedge with simple closure	32	0	0	0
Wedge with patch closure	1	0	0	0
Total laryngectomy with tracheostomy	4	0	0	1 (25.0)
Others	12	1 (8.3)	0	1 (8.3)
Carinal reconstruction	5	0	0	1 (20.0)
Sleeve pneumonectomy	12	0	0	0
Sleeve lobectomy	388	1 (0.3)	0	1 (0.3)
Sleeve segmental excision	15	0	0	0
Bronchoplasty without lung resection	13	0	0	0
Others	29	0	0	0

Values in parenthesis represent mortality %

**Table 46:** **Table 32** 13. Pediatric surgery

	Cases	30 day mortality		Hospital mortality
		Hospital	After discharge	
13. Pediatric surgery	329	2 (0.6)	0	4 (1.2)

Values in parenthesis represent mortality %

**Table 47:** **Table 33** 14. Combined resection of neighboring organ(s)

	Cases	30 day mortality		Hospital mortality
		Hospital	After discharge	
14. Combined resection of neighboring organ(s)	1581	7 (1.4)	3 (0.2)	19 (1.2)
(A) Primary lung cancer (organ resected)				
Aorta	16	0	0	0
Superior vena cava	40	0	0	0
Brachiocephalic vein	12	0	0	0
Pericardium	177	2 (1.1)	0	3 (1.7)
Pulmonary artery	227	1 (0.4)	0	1 (0.4)
Left atrium	45	0	0	1 (2.2)
Diaphragm	98	1 (1.0)	0	1 (1.0)
Chest wall (including ribs)	500	1 (0.2)	0	9 (1.8)
Vertebra	31	0	0	3 (9.7)
Esophagus	12	0	0	0
Total	1158	5 (0.4)	0	18 (1.6)
(B) Mediastinal tumor (organ resected)				
Aorta	3	0	0	0
Superior vena cava	69	0	0	1 (1.4)
Brachiocephalic vein	93	1 (1.1)	0	1 (1.1)
Pericardium	267	1 (0.4)	0	2 (0.7)
Pulmonary artery	9	0	0	0
Left atrium	2	0	0	0
Diaphragm	16	0	0	0
Chest wall (including ribs)	20	0	0	0
Vertebra	7	0	0	0
Esophagus	1	0	0	0
Lung	277	1 (0.4)	0	1 (0.4)
Total	764	3 (0.4)	0	5 (0.7)

Values in parenthesis represent mortality %

**Table 48:** **Table 34** 15. Operation of lung cancer invading the chest wall of the apex

	Cases	30 day mortality		Hospital mortality
		Hospital	After discharge	
15. Operation of lung cancer invading the chest wall of the apex	98	0	0	1 (0.01)

Values in parenthesis represent mortality %

Includes tumors invading the anterior apical chest wall and posterior apical chest wall (superior sulcus tumor, so called Pancoast type)

Interstitial pneumonia was the leading cause of death after lung cancer surgery, followed by pneumonia, respiratory failure, cardiovascular event, and bronchopleural fistula (Table 13).

7829 patients with metastatic pulmonary tumor were operated in 2013 with steady increase similarly to lung cancer surgery (6248, 2009; 6748, 2010; 7210, 2011; 7403, 2012). VATS was adopted in 6323 cases, which comprises 80.8 % of the entire cases. Colo-rectal cancer was by far the leading primary malignancy indicated for resection of metastatic tumors, which comprises 49.8 % of the entire cases (Table 14).

85 tracheal tumors were operated in 2013. Adenoid cystic carcinoma and squamous cell carcinoma were frequent primary tracheal tumors (Table 15).

439 tumors of pleural origin were operated in 2013. Diffuse malignant pleural mesothelioma was the most frequent histology. Extrapleural pneumonectomy was the most frequently chosen operative method (119 cases) with a hospital death of 8.4 % (Table 16).

692 chest wall tumors were resected in 2013 (Table 17).

4780 mediastinal tumors were operated in 2013. There were 2230 thymic epithelial tumors (1904 thymomas, 279 thymic carcinomas, and 47 thymic neuroendocrine carcinoma including carcinoid), followed by 974 congenital cysts, 513 neurogenic tumors, and 243 germ cell tumors. 2624 cases (54.9 %) were resected by VATS (Table 18).

Thymectomy for myasthenia gravis was done in 524 patients, and 271 patients were associated with thymoma, 253 patients were not associated with thymoma. VATS was adopted in 176 cases, which comprises 33.6 % of the entire cases (Table 19).

Lung resection for inflammatory lung diseases were done in 3, 445 patients in 2013. Inflammatory pseudotumor comprised 38.8 % of the entire cases, followed by atypical mycobacterium infection (16.7 %) and fungal infections (13.0 %) (Table 20).

2368 operations for empyema were reported in 2013. There were 1827 patients (77.2 %) with acute empyema and 541 patients with chronic empyema. Bronchopleural fistura was associated in 403 patients (22.1 %) with acute empyema and 287 patients (53.0 %) with chronic empyema. It should be noted that hospital mortality was as high as 10.9 % in patients of acute empyema with fistura (Table 21).

14,612 operations for pneumothorax were reported in 2013. 13,961 operations (95.5 %) were performed by VATS (Table 24).

61 lung transplantations were reported in 2013. Brain-dead donor lung transplantation and living-related donor lung transplantation were done in 41 recipients and 20 recipients, respectively. The number of lung transplantation is still small compared to those in North America and European countries because of shortage of donors (Table 29).

### (C) Esophageal surgery

**Fig. 3:**
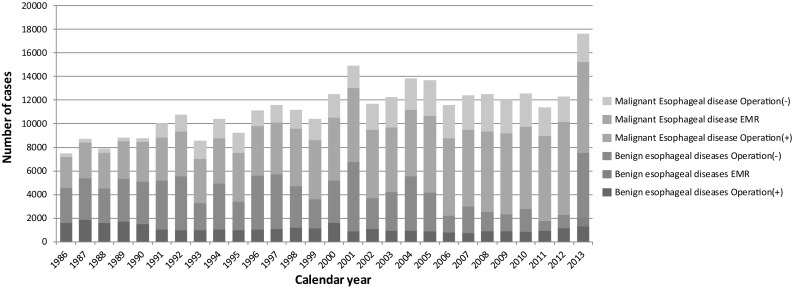
Annual trend of in-patients with esophageal diseases. *EMR* endoscopic mucosal resection (including endoscopic submucosal dissection)

During 2013 alone, a total of 17,656 patients with esophageal diseases were registered from 559 institutions (response rate 96.9 %) which affiliated to the Japanese Association for Thoracic Surgery and/or to the Japan Esophageal Society (Table 1). Among these institutions, those where 20 or more patients underwent esophageal surgeries within the year of 2013 were 186 institutions (33.3 %), which shows no definite shift of esophageal operations to high volume institutions when compared to the data of 2012 (33.2 %) (Table 35). Of 7562 patients with a benign esophageal disease, 1300 (17.2 %) patients underwent surgery, and 761 (10.1 %) patients underwent endoscopic resection, while 5501 (72.7 %) patients did not undergo any surgical treatment (Table 36). Of 10,094 patients with a malignant esophageal tumor, 7677 (76.1 %) patients underwent resection, esophagectomy for 5824 (57.7 %) and endoscopic mucosal resection (EMR) or endoscopic submucosal dissection (ESD) for 1853 (18.4 %), while 2417 (23.9 %) patients did not undergo any resection (Tables 37, 38). The increase of registered patients with endoscopic resection and nonsurgically treated benign esophageal diseases is obvious during 2012 and 2013. The patients registered, particularly those undergoing ESD or EMR and nonsurgical therapy for a malignant esophageal disease, have been increasing since 1990 (Fig. 3).

**Table 49:** **Table 35** Distribution of number of esophageal operations in 2013 in each institution

Esophageal surgery			
Number of operations in 2013	Benign esophageal diseases	Malignant esophageal disease	Benign + malignant
*0*	268	116	84
1–4	196	108	96
5–9	51	69	84
10–19	23	104	109
20–29	8	47	56
30–39	6	33	35
40–49	2	19	26
≧50	5	63	69
Total	559	559	559

**Table 50:** **Table 36** Benign esophageal diseases

	Operation (+)												Endoscopic resection	Operation (−)	Total
	Number of patients			30-day mortality						Hospital mortality					
	Total	Open	T/L*3	Open Surgery			T/L*3			Total	Open surgery	T/L*3			
				Total	Hospital	After discharge	Total	Hospital	After discharge						
1. Achalasia	322	203	119	0	0	0	0	0	0	0	0	0		31	353
2. Benign tumor	90	57	33	0	0	0	2 (6.1)	0	2 (6.1)	3 (3.3)	1 (1.8)	2 (6.1)	283	36	409
(1) Leiomyoma	60	36	24	0	0	0	1 (4.2)	0	1 (4.2)	1 (1.7)	0	1 (4.2)	13	32	105
(2) Cyst	5	4	1	0	0	0	0	0	0	0	0	0	1	0	6
(3) Others	25	17	8	0	0	0	1 (12.5)	0	1 (12.5)	2 (8.0)	1 (5.9)	1 (12.5)	268	4	297
(4) Not specified	0	0	0	0	0	0	0	0	0	0	0	0	1	0	1
3. Diverticulum	30	25	5	0	0	0	0	0	0	0	0	0		45	75
4. Hiatal hernia	509	390	119	1 (0.3)	1 (0.3)	0	0	0	0	6 (1.2)	5 (1.3)	1 (0.8)		1948	2457
5. Spontaneous rupture of the esophagus	112	100	12	2 (2.0)	2 (2.0)	0	0	0	0	5 (4.5)	5 (5.0)	0		13	125
6. Esophago-tracheal fistula	16	16	0	1 (6.3)	1 (6.3)	0	0	0	0	1 (6.3)	1 (6.3)	0		5	21
7. Congenital esophageal atresia	48	42	6	0	0	0	0	0	0	1 (2.1)	1 (2.4)	0		3	51
8. Congenital esophageal stenosis	10	9	1	0	0	0	0	0	0	0	0	0		5	15
9. Corrosive stricture of the esophagus	8	7	1	0	0	0	0	0	0	0	0	0		6	14
10. Esophagitis, esophageal ulcer	28	23	5	0	0	0	0	0	0	0	0	0		411	439
11. Esophageal varices	62	61	1	4 (6.6)	4 (6.6)	0	0	0	0	6 (9.7)	6 (9.8)	0		2860	2922
(1) Laparotomy	15	14	1	0	2 (14.3)	0	0	0	0	3 (20.0)	3 (21.4)	0			15
(2) Sclerotherapy				0		0	0	0	0	0			407	283	690
12. Others	65	56	9	3 (5.4)	2 (3.6)	0	0	0	0	7 (10.8)	5 (8.9)	2 (22.2)	71	138	274
Total	1300	989	311	10 (1.0)	10 (1.0)	0	2 (0.6)	0	2 (0.6)	29 (2.2)	24 (2.4)	5 (1.6)	761	5501	7562

Values in parenthesis represent mortality %

*T/L* thoracoscopic and/or laparoscopic

**Table 51:** **Table 37** Malignant esophageal diseases (histologic classification)

	Resection (+)	Resection (−)	Total
Carcinomas	7616	2388	10,004
1. Squamous cell carcinoma	6836	2279	9115
2. Basaloid(-squamous) carcinoma	68	8	76
3. Carcinosarcoma	36	4	40
4. Adenocarcinoma in the Barrett’s esophagus	306	26	332
5. Other adenocarcinoma	281	32	313
6. Adenosquamous carcinoma	28	2	30
7. Mucoepidermoid carcinoma	9	0	9
8. Adenoid cystic carcinoma	1	0	1
9. Endocrine cell carcinoma	35	22	57
10. Undifferentiated carcinoma	4	7	11
11. Others	12	8	20
Other malignancies	41	3	44
1. Malignant non-epithelial tumors	14	1	15
2. Malignant melanoma	22	2	24
3. Other malignant tumors	5	0	5
Not specified	20	26	46
Total	7677	2417	10,094

Resection: including endoscopic resection

**Table 52:** **Table 38** Malignant esophageal disease (clinical characteristics)

	Operation (+)					EMR or ESD	Operation (−)	Total
	Cases	30-day mortality			Hospital mortality			
		Total	Hospital	After discharge				
1. Esophageal cancer	5824	41 (0.7)	41 (0.7)	0	114 (2.0)	1853	2417	10,094
Location								0
(1) Cervical esophagus	195	0	0	0	3 (1.5)	80	178	453
(2) Thoracic esophagus	4758	32 (0.7)	32 (0.7)	0	98 (2.1)	1332	1847	7937
(3) Abdominal esophagus	605	6 (1.0)	6 (1.0)	0	9 (1.5)	70	66	741
(4) Multiple cancers	262	2 (0.8)	2 (0.8)	0	3 (1.1)	130	59	451
(5) Others/not described	4	1 (25.0)	1 (25.0)	0	1 (25.0)	241	267	512
Tumor depth								
(A) Superficial cancer (T1)	1799	8 (0.4)	8 (0.4)	0	19 (1.1)	1757	192	3748
*Mucosal cancer* (*T1a*)	*425*	*1* (*0.2*)	*1* (*0.2*)	*0*	*2* (*0.5*)	*1285*	*24*	*1734*
(B) Advanced cancer (T2–T4)	4025	33 (0.8)	33 (0.8)	0	94 (2.3)	1	2198	6224
(C) Not specified	0	0	0	0	0	95	27	122
2. Multiple primary cancers	984	6 (0.6)	6 (0.6)	0	19 (1.9)	355	323	1662
1) Synchronous	553	4 (0.7)	4 (0.7)	0	11 (2.0)	146	174	873
(1) Head and neck	166	0	0	0	0	67	50	283
(2) Stomach	197	1 (0.5)	1 (0.5)	0	2 (1.0)	43	65	305
(3) Others	157	3 (1.9)	3 (1.9)	0	8 (5.1)	23	49	229
(4) Triple cancers	24	0	0	0	0	13	6	43
(5) Unknown	9	0	0	0	1 (11.1)	0	4	13
2) Metachronous	428	2 (0.5)	2 (0.5)	0	8 (1.9)	209	149	786
(1) Head and neck	94	1 (1.1)	1 (1.1)	0	0	63	40	197
(2) Stomach	103	0	0	0	2 (1.9)	59	31	193
(3) Others	197	1 (0.5)	1 (0.5)	0	4 (2.0)	59	63	319
(4) Triple cancers	32	0	0	0	1 (3.1)	28	12	72
(5) Unknown	2	0	0	0	1 (50.0)	0	3	5
Unknown	3	0	0	0	0	0	0	0

Values in parenthesis represent mortality %

*EMR* endoscopic mucosal resection (including endoscopic submucosal dissection)

Among benign esophageal diseases (Table 36), hiatal hernia, esophageal varices, esophagitis (including reflux esophagitis) and achalasia were the most common conditions in Japan. On the other hand, spontaneous rupture of the esophagus, benign esophageal tumors and congenital esophageal atresia were common diseases which were surgically treated as well as the above-mentioned diseases. The thoracoscopic and/or laparoscopic procedures have been widely adopted for benign esophageal diseases, in particular achalasia, hiatal hernia and benign tumors. Open surgery was performed in 989 patients with a benign esophageal disease, with 30-day mortality in 10 (1.0 %), while thoracoscopic and/or laparoscopic surgery was performed for 311 patients, with 2 (0.6 %) of the 30-day mortality The difference in these death rates between open and scopic surgery seem to be related the conditions requiring open surgery.

The majority of malignant diseases were carcinomas (Table 37). Among esophageal carcinomas, the incidence of squamous cell carcinoma was 91.1 %, while that of adenocarcinomas including Barrett cancer was 6.4 %. The resection rate for patients with a squamous cell carcinoma was 75.0 %, while that for patients with an adenocarcinoma was 91.0 %.

According to location, cancer in the thoracic esophagus was the most common (Table 38). Of the 3748 patients (37.1 % of total esophageal malignancies) having superficial esophageal cancers within mucosal and submucosal layers, 1799 (48.0 %) patients underwent esophagectomy, while 1757 (46.9 %) patients underwent EMR or ESD. The 30-day mortality rate and hospital mortality rate after esophagectomy for patients with a superficial cancer were 0.2 and 0.5 % respectively. Advanced esophageal cancer invading deeper than the submucosal layer was observed in 6224 (61.7 %) patients. Of the 6224 patients with advanced esophageal cancer, 4025 (64.7 %) underwent esophagectomy, with 0.8 % of the 30-day mortality rate, and with 2.3 % of the hospital mortality rate.

Multiple primary cancers were observed in 1662 (16.5 %) of all the 10,094 patients with esophageal cancer. Synchronous cancer was found in 873 (52.5 %) patients, while metachronous cancer (found before esophageal cancer) was observed in 786 (47.3 %) patients. The stomach is the commonest site for both synchronous and metachronous malignancy followed by head and neck cancer (Table 38).

**Fig. 4:**
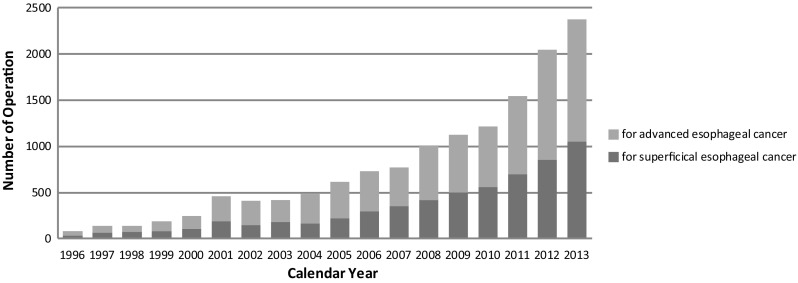
Annual trend of video-assiste d esophagectomy for esophageal malignancy

Among esophagectomy procedures, transthoracic esophagectomy through right thoracotomy was the most commonly adopted for patients with a superficial cancer as well as for those with an advanced cancer (Table 39). Transhiatal esophagectomy commonly performed in Western countries was adopted in only 4.6 % of patients having a superficial cancer who underwent esophagectomy and in 1.5 % of those having an advanced cancer in Japan. The thoracoscopic and/or laparoscopic esophagectomy were adopted for 1049 patients (58.3 %) with a superficial cancer, and for 1326 patients (32.9 %) with an advanced cancer. The number of cases of thoracoscopic and/or laparoscopic surgery for superficial or advanced cancer has been increasing for these several years (Fig. 4).

**Table 53:** **Table 39** Malignant esophageal disease (surgical procedures)

	Cases	Operation (+)			Thoracoscopic and/or laparoscopic procedure				EMR or ESD
		30-day mortality		Hospital mortality	Cases	30-day mortality		Hospital mortality	
		Hospital	After discharge			Hospital	After discharge		
Superficial cancer (T1)	*1799*	*8* (*0.4*)	*0*	*19* (*1.1*)	*1049*	*1* (*0.1*)	*0*	*6* (*0.6*)	1757
*Mucosal cancer* (*T1a*)	*425*	*1* (*0.2*)	*0*	*2* (*0.5*)	*212*	*0*	*0*	*0*	*1285*
Esophagectomy	*1799*	*8* (*0.4*)	*0*	*19* (*1.1*)	*1049*	*1* (*0.1*)	*0*	*6* (*0.6*)	1757
(1) Transhiatal esophagectomy	83	0	0	1 (1.2)	18	0	0	0	
(2) Transthoracic (rt.) esophagectomy and reconstruction	1451	6 (0.4)	0	15 (1.0)	886	1 (0.1)	0	5 (0.6)	
(3) Transthoracic (lt.) esophagectomy and reconstruction	65	0	0	0	20	0	0	0	
(4) Cervical esophageal resection and reconstruction	9	0	0	0	2	0	0	0	
(5) Two stage operation	18	1 (5.6)	0	2 (11.1)	12	0	0	1 (8.3)	
(6) Others	119	0	0	0	108	0	0	0	
(7) Not specified	54	1 (1.9)	0	1 (1.9)	3	0	0	0	
Advanced cancer (T2–T4)									
Esophagectomy	*4025*	*33* (*0.8*)	*0*	*94* (*2.3*)	*1326*	*14* (*1.1*)	*0*	*35* (*2.6*)	1
(1) Transhiatal esophagectomy	60	1 (1.7)	0	4 (6.7)	6	0	0	0	
(2) Transthoracic (rt.) esophagectomy and reconstruction	3340	25 (0.7)	0	69 (2.1)	1173	11 (0.9)	0	29 (2.5)	
(3) Transthoracic (lt.) esophagectomy and reconstruction	158	3 (1.9)	0	3 (1.9)	4	1 (25.0)	0	1 (25.0)	
(4) Cervical esophageal resection and reconstruction	105	0	0	2 (1.9)	5	0	0	0	
(5) Two stage operation	97	1 (1.0)	0	10 (10.3)	20	0	0	2 (10.0)	
(6) Others/not specified	241	3 (1.2)	0	6 (2.5)	114	2 (1.8)	0	3 (2.6)	
(7) Not specified	24	0	0	0	4	0	0	0	
(Depth not specified)	*0*	*0*	*0*	*0*	*5*	*0*	*0*	*1* (20.0)	95
Combined resection of other organs	*281*	*4* (*1.4*)	*0*	*8* (*2.8*)					
(1) Aorta	2	0	0	0					
(2) Trachea, bronchus	44	0	0	1 (2.3)					
(3) Lung	77	1	0	4 (5.2)					
(4) Others	156	3 (1.9)	0	3 (1.9)					
Unknown	2	0	0	0					
Salvage surgery	*234*	*2* (*0.9*)	*0*	*14* (*6.0*)	*38*	*0*	*0*	*2* (*5.3*)	34

Values in parenthesis represent mortality %

Combined resection of the neighboring organs during resection of an esophageal cancer was performed in 281 patients (Tables 39, 40). Resection of the aorta together with the esophagectomy was performed in 2 cases. Tracheal and/or bronchial resection combined with esophagectomy was performed in 44 patients, with the 30-day mortality rate at 0 % and the hospital mortality rate at 2.3 %. Lung resection combined with esophagectomy was performed in 77 patients, with the 30-day mortality rate at 1.3 % and the hospital mortality rate at 5.2 %.

**Table 54:** **Table 40** Mortality after combined resection of the neighboring organs

Year	Esophagectomy			Combined resection											
				Aorta			Tracheobronchus			Lung			Others		
	a	b	c (%)	a	b	c (%)	a	b	c (%)	a	b	c (%)	a	b	c (%)
1996	4194	120	2.86	7	3	42.86	24	0	0.00	50	2	4.00	78	4	5.13
1997	4441	127	2.86	1	0	0.00	34	5	14.71	56	1	1.79	94	3	3.19
1998	4878	136	2.79	4	0	0.00	29	0	0.00	74	1	1.35	128	2	1.56
1999	5015	116	2.31	5	0	0.00	23	2	8.70	68	0	0.00	122	1	0.82
2000	5350	81	1.51	2	0	0.00	23	2	8.70	69	0	0.00	96	1	1.04
2001	5521	110	1.99	1	0	0.00	26	1	3.85	83	3	3.61	99	2	2.02
2002	4904	66	1.35	3	1	33.33	20	2	10.00	63	0	0.00	63	1	1.59
2003	4639	45	0.97	0	0	0.00	24	2	8.33	58	0	0.00	88	1	1.14
2004	4739	64	1.35	2	0	0.00	17	0	0.00	59	5	8.47	119	2	1.68
2005	5163	52	1.01	1	0	0.00	11	1	9.09	67	1	1.49	73	1	1.37
2006	5236	63	1.20	0	0	0.00	17	0	0.00	62	2	3.23	122	3	2.46
2007	4990	60	1.20	0	0	0.00	25	1	4.00	44	1	2.27	138	2	1.45
2008	5124	63	1.23	0	0	0.00	17	1	5.88	48	1	2.08	185	0	0.00
2009	5260	63	1.20	0	0	0.00	19	2	10.53	58	2	3.45	211	3	1.42
2010	5180	45	0.87	2	0	0.00	33	0	0.00	58	0	0.00	245	5	2.04
2011	5430	38	0.70	4	0	0.00	26	0	0.00	41	0	0.00	179	5	2.79
2012	6055	47	0.78	2	0	0.00	23	1	4.35	69	0	0.00	240	1	0.42
2013	5824	41	0.70	2	0	0.00	44	0	0.00	77	1	1.30	156	3	1.92
Total	91,943	1040	1.13	26	4	15.38	273	16	5.86	753	16	2.12	1220	23	1.89

*a* number of patients who underwent the operation, *b* number of patients died within 30 days after operation, *c* % ratio of b/a, *i.e.*, direct operative mortality

Salvage surgery after definitive (chemo-) radiotherapy was performed in 234 patients, with the 30-day mortality rate at 0.9 % and with the hospital mortality rate at 6.0 % (Table 39).

Lastly, in spite of the efforts of the Committee to cover wider patient populations to this annual survey, the majority of the institutions which responded to the questionnaire were the departments of thoracic or esophageal surgery. It should be noted that larger number of patients with esophageal diseases should have been treated medically and endoscopically. We should continue our effort for complete survey through more active collaboration with the Japan Esophageal Society and other related societies.

